# Reconstructing NOD-like receptor alleles with high internal conservation in Podospora anserina using long-read sequencing

**DOI:** 10.1099/mgen.0.001442

**Published:** 2025-07-02

**Authors:** S. Lorena Ament-Velásquez, Brendan Furneaux, Sonia Dheur, Alexandra Granger-Farbos, Rike Stelkens, Hanna Johannesson, Sven J. Saupe

**Affiliations:** 1 Department of Zoology, Stockholm University, 106 91 Stockholm, Sweden; 2 Department of Biological and Environmental Science, University of Jyväskylä, 40014 Jyväskylä, Finland; 3 IBGC UMR 5095 CNRS University of Bordeaux, 33077 Bordeaux, France; 4 Department of Ecology, Environmental and Plant Sciences, Stockholm University, 106 91 Stockholm, Sweden; 5 The Royal Swedish Academy of Sciences, 114 18 Stockholm, Sweden

**Keywords:** allorecognition, fungi, heterokaryon incompatibility, WD40 domain

## Abstract

NOD-like receptors (NLRs) are intracellular immune receptors that detect pathogen-associated cues and trigger defence mechanisms, including regulated cell death. In filamentous fungi, some NLRs mediate heterokaryon incompatibility, a self-/non-self-recognition process that prevents the vegetative fusion of genetically distinct individuals, reducing the risk of parasitism. The *het-d* and *het-e* NLRs in *Podospora anserina* are highly polymorphic incompatibility genes (*het* genes) whose products recognize different allelic variants of the HET-C protein via a sensor domain composed of WD40 repeats. These repeats display unusually high sequence identity maintained by concerted evolution. However, some sites within individual repeats are hypervariable and under diversifying selection. Despite extensive genetic studies, inconsistencies in the reported WD40 domain sequence have hindered functional and evolutionary analyses. Here, we confirm that the WD40 domain can be accurately reconstructed from long-read sequencing (Oxford Nanopore and PacBio) data, but not from Illumina-based assemblies. Functional alleles are usually formed by 11 highly conserved repeats, with different repeat combinations underlying the same phenotypic *het-d* and *het-e* incompatibility reactions. AlphaFold 3 structure models suggest that their WD40 domain folds into two 7-blade *β*-propellers composed of the highly conserved repeats, as well as three cryptic divergent repeats at the C-terminus. We additionally show that one particular *het-e* allele does not have an incompatibility reaction with common *het-c* alleles, despite being 11-repeats long. Finally, we present evidence that the recognition phenotypes of *het-e* and *het-d* arose through convergent evolution. Our findings provide a robust foundation for future research into the molecular mechanisms and evolutionary dynamics of *het* NLRs, while also highlighting both the fragility and the flexibility of *β*-propellers as immune sensor domains.

**Impact Statement**

Innate immunity relies on specialized receptors to detect pathogens and trigger defence responses. NOD-like receptors (NLRs) are a class of intracellular receptors conserved across all domains of life including plants, animals, fungi and bacteria. A striking characteristic of fungal NLRs is the fact that a fraction of them display C-terminal sensor domains composed of highly similar tandem repeats. The *het-d* and *het-e* genes in the model fungus *Podospora anserina* are classic examples of this NLR architecture. Although their genetics are well characterized, inconsistencies in their reported sequences have hindered functional and evolutionary analyses. By comparing genome assemblies generated with different sequencing technologies from the same strains, we confirm that short-read assemblies of these NLRs are unreliable. Using long-read sequencing, we accurately reconstruct the sensor domains of multiple alleles and reveal that different repeat combinations can lead to the same binding specificities. Using AlphaFold, we further provide a model for the overall structural organization of their sensor domains. More broadly, the assembly challenges and structural features of the *het-d* and *het-e* NLRs likely extend to other fungal NLRs with similar repeat architectures.

## Data Summary

All sequencing data generated in this study has been submitted to the NCBI BioProject database (https://www.ncbi.nlm.nih.gov/bioproject/) under accession numbers SRR32142581–SRR32142604 (BioProject PRJNA1216259). Associated genome assemblies have been submitted to the Dryad Digital Repository (https://doi.org/10.5061/dryad.h18931zww). Previously published long-read genome assemblies used here are also available in the Dryad Digital Repository (https://doi.org/10.5061/dryad.1vhhmgr0j). The snakemake pipeline used for WD40 repeat classification and all the nt sequences of the *het* genes (aligned and in fasta format) are available at https://github.com/SLAment/FixingHetDE.

## Introduction

NOD-like receptors (NLRs) are a class of almost universally conserved intracellular immune receptors that play crucial roles in animal, plant, fungal and bacterial host defence systems [1,4]. Sometimes referred to as cellular ‘guardians’, NLRs can sense cues of the unwanted invasion of non-self entities, such as pathogen-derived molecules or pathogen-induced modifications of host cells [5]. Typically, NLRs have a tripartite domain architecture and function through ligand-induced oligomerization [6,8]. When a non-self ligand binds to the C-terminal domain (the ‘sensor’), which is normally composed of superstructure-forming repeats, it triggers the multimerization of the central nt-binding and oligomerization domain (NBD). This change in the NBD, in turn, activates the N-terminal effector domain that usually leads to regulated cell death [9]. Given this general mode of action, the sensor domain can be under strong selective pressure to keep up with the evolution of pathogens, which change constantly to avoid detection [4,10, 11].

Filamentous fungi possess large and diverse repertoires of NLRs [1,12, 13]. However, only a few have been functionally characterized, all within the context of heterokaryon or vegetative incompatibility – a self-/non-self-recognition mechanism occurring between strains of the same species [14]. Growth in filamentous fungi is accomplished by extending their cells or hyphae, by branching and by fusing with other cells, leading to the possibility of fusing with other individuals [15,16]. This vegetative fusion with non-self poses a great risk, since it opens the door for intracellular parasites such as mycoviruses and selfish organelles, including nuclei [17,20]. As a form of defence, different individuals can fuse successfully only if they are compatible at a set of specific loci, termed heterokaryon incompatibility (*het*) genes, some of which are NLRs. Mirroring the innate immune response of other eukaryotes and bacteria, the *het* NLRs trigger regulated cell death of the fused incompatible hyphae, preventing the exchange of cytoplasm and hence parasites [21]. In plate cultures, this phenomenon can be observed as a line of dead cells in the contact zone between two incompatible strains, called the ‘barrage’ [22]. In accordance with their self-/non-self-recognition function, *het* genes in general are highly polymorphic at the population level, displaying signatures of balancing selection [23,25].

**Fig. 1.**
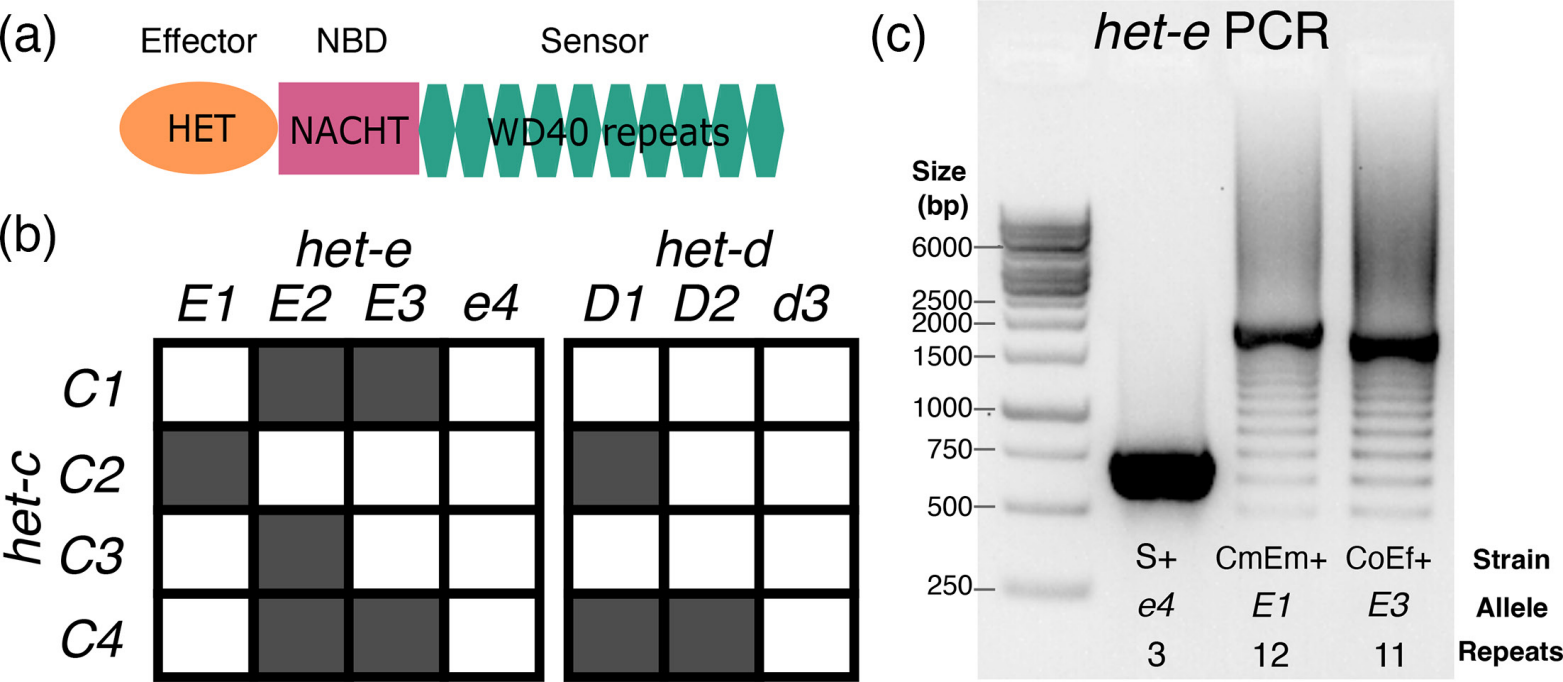
Primer on the *het-d* and *het-e* genes. (**a**) The domain structure of an HNWD NLR. (**b**) Incompatibility interactions between the most common *het-c* alleles and those of *het-e* and *het-d*. Shaded squares indicate a vegetative incompatibility reaction, while white squares indicate compatibility, following Saupe *et al*. [38]. (**c**) A typical PCR result when amplifying the WD40 domain of an HNWD gene from genomic DNA, in this case *het-e* (1% agarose gel). Three strains with known *het-e* alleles are shown.

Among filamentous fungi, *Podospora anserina* has one of the best-studied repertoires of *het* genes [22,26]. Early classical genetic work on a collection of 16 strains collected in France determined the existence of 9 *het* loci [27,28], all of which have now been cloned (reviewed in [29]). From these genes, *het-r*, *het-d* and *het-e* are paralogues from the same NLR type, collectively known as HNWD genes based on their domain architecture [30]. Specifically, HNWD genes are characterized by having a TIR-related HET effector domain at the N-terminus, an NBD of the NACHT type, and a sensing domain formed by WD40 repeats at the C-terminus (Fig. 1a). WD40 domains in general form doughnut-like (toroidal) folds called *β*-propellers assembled from six to eight repeats [31]. While many NLRs have WD40 domains, the HNWD sensor domain is peculiar in several aspects. On the one hand, the individual WD40 repeats display high sequence identity, ranging from over 80% to 100% within each gene [30,32, 33]. It is proposed that such a level of high internal conservation (HIC) reflects the concerted evolution of the repeats through unequal crossing-overs or other recombination events that cause high allelic variability [30,34, 35]. This process can add or remove repeat units, leading to length polymorphism in natural populations. On the other hand, while being overall very similar, the individual repeats also show extensive variability at four specific codon positions under diversifying selection, which map to aa residues predicted at the interaction surface of the *β*-propeller [30].

Previous research has shown that the number and sequence of WD40 repeats determine the allele specificity of a given HNWD paralogue [32,35,37]. For example, the product of one specific *het-r* allele of 11 repeats (also known as *het-R* or just *R*) recognizes one allele of the *het-v* locus, triggering the vegetative incompatibility reaction [35]. Other combinations and numbers of repeats are not reactive (known as *r*). Meanwhile, the products of the *het-d* and *het-e* genes recognize the same target, the glycolipid transfer protein coded by the *het-c* gene [28,38]. The *het-c* gene itself is polymorphic, and its different (phenotypic) alleles can be defined by their interaction with *het-d* and *het-e* [28]. For instance, the *C2* allele triggers an incompatibility reaction with a specific *het-e* allele (*E1*) and also with one particular *het-d* allele (*D1*), but not with other alleles (Fig. 1b). In other words, the other *het-d/e* alleles do not recognize *C2* as a ligand. To date, three alleles of *het-e* (*E1*, *E2* and *E3*) are known to recognize *het-c*, while null variants are collectively known as the *e4* allele. Likewise, *het-d* has two known reactive alleles (*D1* and *D2*) and a non-reactive allele (*d3*). (In the literature, the different *het* alleles might be referred to as *het-E1*, *het-E2*, etc., but here, we use a simplified terminology for readability.)

Although the genetics of *het-d* and *het-e* are well understood, the precise characteristics of their sensor domain remain unclear, likely due to their repetitive nature and HIC properties. The original study that identified *het-e* sequenced the allele of the French strain A (here referred to as *E1*^A^) and reported ten WD40 repeats [32]. Later, Espagne *et al*. [33] resequenced the same *E1*^A^ allele but found differences in multiple aa. They also sequenced the *het-e* allele of strain C (*E2*^C^, ten repeats) and used PCR and Southern blotting to estimate the number of repeats from several WT and mutant strains from the original French collection. On occasion, these two methods returned conflicting results, in which case they gave priority to the Southern blot analysis since PCR is susceptible to amplification artefacts (Fig. 1c). Overall, they concluded that at least ten repeats are necessary for *het-e* to be reactive, that losing even a single repeat can break an allele and that some alleles have the right size but are still non-reactive (*e4*) [32,33]. More recently, Chevanne *et al*. [35] resequenced *E1*^A^ yet again and found it to contain 11 repeats instead. In the case of *het-d*, only a single allele has been sequenced, *D2*^Y^ (from the French strain Y), consisting of 11 full repeats and the first 30 aa of a twelfth repeat at the C-terminus [33]. As for *het-e*, Espagne *et al*. [33] examined the sizes of French WT *het-d* alleles by PCR and Southern blot, inferring that the functional *D1*^F^ also has 11 full repeats and a truncated one, while non-reactive alleles (*d3*) have either less or more repeats. Thus, the actual sequences of most active alleles remain unknown, precluding additional functional and evolutionary studies. Moreover, the reported sizes of functional HNWD alleles (e.g. 10 or 11) are at odds with the number of repeats expected from usual *β*-propellers, which is 6 to 8 repeats but most often 7 [39].

The growing availability of genomic data has thrust the study of fungal NLRs into new frontiers [1,12, 37]. However, modern whole-genome sequencing using short-read (Illumina) technologies is not necessarily the solution for NLRs with HIC: tandem repeats with high sequence similarity can be notoriously difficult to assemble [40]. The length of a single WD40 repeat is 126 bp (42 aa), close to the size of a typical Illumina read, making it a borderline case. Long-read technologies, such as PacBio or Oxford Nanopore Technologies (ONT), hold the promise of accurate genome assembly, especially as error rates and costs decrease [41,42]. Here, we took advantage of published Illumina, PacBio and ONT datasets of WT *P. anserina* strains [43,44] to examine the HNWD alleles in the context of different sequencing technologies and assembly software. To resolve inconsistencies in the literature, we produced new ONT data to recover the reactive *het-d* and *het-e* alleles of the original French strains. Having established a reliable set of HNWD sequences, we assessed the interactions of a *het-e* allele seen in several WT strains. We discuss possible arrangements of their *β*-propeller domain using AlphaFold 3 protein structure models. Finally, we explore the phylogenetic relationships of the different domains in HNWD genes and related NLRs. Overall, we provide a basis for the study of the binding specificity and evolution of these variable immune receptors.

## Methods

### Fungal material and culture conditions

The strains used in this study were obtained from either the University of Bordeaux [32] or from the collection maintained in the Johannesson Lab at Stockholm University, which in turn came from the Laboratory of Genetics at Wageningen University [43,45]. Work with all strains was done using monokaryotic (haploid) isolates, including those corresponding to the sequenced monokaryons in Vogan *et al*. [43,44]. Hence, strains are designated by their name and their mating type (e.g. Wa63+ is the Wageningen Collection strain 63 with a mating type +).

Mycelia for DNA extraction was obtained from two sources: Petri dishes (strains Y+, Wa63+ and Z+) and liquid cultures (all the strains with introgressed *het-c*, *het-d* and *het-e* alleles into the strain s background, see below). The cultures on Petri dishes were done with HPM media [43] plates topped with cellophane discs cut from X50 Cellophane membrane 14×14 cm sheets (Fisher Scientific GTF AB, product code 11927535) and previously autoclaved in deionized water between filter paper discs [46]. Plates were incubated at 27 °C under 70% humidity for a 12:12 light:dark cycle for 2 or 3 days (if left longer, the mycelia ages and becomes harder to remove from the cellophane). Around 100 mg of mycelia was harvested by scraping the cellophane disc with a cell scraper (Sarstedt, Inc., 83.3951) and stored at −70 °C.

It has been reported that *P. anserina* cultures in Luria–Bertani (LB) broth do not undergo senescence and are appropriate to get abundant and healthy mycelia [47]. We tested the use of both LB agar (LA) plates and LB cultures for mycelia harvesting. We found that the growth in LA or LB media significantly varies depending on the *P. anserina* strain. While strains S (used by Benocci *et al*. [47]) and s thrive, some strains from the Wageningen collection exhibit poor growth. We also found that LA plates are not appropriate for the mid-term storage of strains. Hence, we used LB cultures just for the DNA extraction of the *het*-gene introgressed strains. Specifically, we used a modified LB recipe from Benocci *et al*. [47] that contains 10 g l^−1^ tryptone, 5 g l^−1^ yeast extract, 5 g l^−1^ NaCl and 0.02 g l^−1^ thymine 99%, to which we added biotin and thiamine to a final concentration of 5 and 100 µg l^−1^, respectively, and 1 ml l^−1^ of the trace element solution of [48]. We cut pieces of agar with mycelium from PASM0.2 plates [48] grown as for the HPM plates above and used them as inocula for flasks containing 200 ml of modified LB. We incubated the flasks at 27 °C and 120 r.p.m. for 5 days [43]. The resulting mycelia balls were recovered from the flask with sterilized tweezers and stored at −70 °C before extraction.

### DNA extraction and sequencing

Whole-genome DNA was extracted with the Zymo Quick-DNA Fungal/Bacterial Miniprep Kit D6005 (Zymo Research; https://zymoresearch.eu/) and quantified with a Qubit 2.0 Fluorometer (Invitrogen). For the strain CmEm−, ~800 mg of mycelia was used for high-molecular-weight (HMW) DNA extraction using the QIAGEN Genomic-tip 100 G^−1^ kit (Qiagen).

ONT sequencing was performed in-house using a Native Barcoding Kit 24 V14 SQK-NBD114.24 and a MinION Mk1C machine following the standard protocol. In total, 12 strains were barcoded into 2 pools (pool1: CmEm−, CoEc+, CoEc−, Y+, Z+ and Wa63+ with barcodes 1 to 6 and pool2: CoEf+, ChEhDa+, ChEhDa−, CaDa−, CsDf+ and CsDf− with barcodes 7 to 12). Each pool was sequenced in two separate R10.4.1 flow cells (FLO-MIN114), aiming at loading around 10–20 fmol of the library for optimal duplex output (while assuming a highly fragmented DNA extraction to the detriment of sample CmEm-). Both libraries included 1 µl of diluted DNA control sample (DNA CS), a 3.6 kb standard amplicon used to do quality control on the library. We added 5 µl of BSA (Invitrogen UltraPure BSA 50 mg ml^−1^, AM2616) to the flow cell priming mix as recommended. The 4 flow cells (first 2 for pool1 and last 2 for pool2) were run until about 50 pores remained active (for 41–54 h), generating 10.09, 9.89, 9.19 and 9.06 Gb estimated bases, respectively. All runs had the following settings: pore scan frequency of 1.5 h, minimum read length of 200 bp, read splitting on and active channel selection on. The strains CoEc−, ChEhDa− and CsDf− yield identical results to their opposite mating type counterparts, so they were not discussed further in this study.

PCR amplification of *het-e* in Fig. 1 was done with the forward 5′-GCCCTTGTATTTGCACCGAC-3′ and reverse 5′-CGTCCTGAGTAACAGCCAAGAAC-3′ primers, using the following temperature regime: 95 °C for 1 min; 35 cycles at 95 °C for 15 s, 64 °C for 15 s and 72 °C for 30 s; and 72 °C for 7 min. The PCR reaction contained 8 µl ddH20, 0.5 µl of each primer (10 µM), 1 µl of sample DNA and 10 µl of MyTaq Red Mix (Meridian Bioscience™) for a total volume of 20 µl.

### Basecalling

During sequencing in the MinION Mk1C machine, we activated the ‘Fast model, 400 bps’ for live basecalling with Guppy v7.1.4 (embedded in MinKNOW v23.07.12). These reads were used only for preliminary coverage assessment per sample and automatic demultiplexing. The demultiplexed pod5 files were basecalled using Dorado v0.5.3 (https://github.com/nanoporetech/dorado/) with the dna_r10.4.1_e8.2_400bps_sup@v4.3.0 model. The resulting BAM files were transformed into fastq files with the bam2fq program of SAMtools v1.19.2 [49]. Reads corresponding to the DNA CS introduced during library preparation were removed using chopper v0.7.0 [50].

### Genome assembly and sequence analyses

For each sample, we removed reads that contained perfect matches to ONT native barcodes assigned to other samples (0.06%–0.26% of the reads). We removed barcodes and performed minimum quality control with fastplong v0.2.2 [51] and parameters --trimming_extension 20 -l 50 -q 15 -d 0.1 (hereafter, cleaned ONT reads). The cleaned ONT reads of each sample were used as input for Flye v2.9.3 [52], with parameters --nano-hq --iterations 2. The scaffolds were oriented to match the chromosomes of the reference genome Podan2 [53]. We visually looked for major rearrangements by mapping all assemblies to Podan2 with the NUCmer program of MUMmer v3.23 [54]. The Integrative Genomics Viewer (IGV) browser was used for read-mapping visualization [55]. The median read length and depth of coverage of the ONT R10 datasets were estimated by mapping the cleaned reads to their respective assemblies using minimap2 v2.26 [56] and feeding the produced BAM file to Cramino v0.14.1 [50]. Equivalent values for published long-read assemblies of Wa63+ and Y+ genomes were taken from Vogan *et al*. [43].

The paired-end Illumina reads of the strains Wa63+, Y+, Z+ and Wa137- were retrieved from NCBI’s Sequence Read Archive (accession numbers SRX5458088, SRX5458091, SRX11405146 and SRX8537866) and assembled with SPAdes v4.0.0 [57] using the --careful parameter and either the default k-mer setting (Wa63−, Z+ and Y−) or the k-mers 21, 33, 55 and 77 (all strains). The Illumina reads were mapped back to each assembly using BWA v0.7.18 [58]. The resulting BAM file was sorted with SAMtools v1.21 [49], and the duplicates were marked with Picard v3.3.0 (http://broadinstitute.github.io/picard/) with a value of 100 (Wa63- and Y-) or 2,500 (Wa137-) for the --OPTICAL_DUPLICATE_PIXEL_DISTANCE parameter. Finally, the deduplicated BAM file was given as input of Qualimap v2.2.2d [59] to obtain the average depth of coverage per assembly.

The nt sequences of *het-d*, *het-e* and *het-r* were extracted from each assembly using the script query2haplotype.py v2.22 available at https://github.com/SLAment/Genomics with parameters --haplo --extrabp 800 --minsize 400 --vicinity 15000 --identity 95 and the S+ allele as query. The sequences were manually aligned and given as input for a custom snakemake pipeline for WD40 repeat classification (https://github.com/SLAment/FixingHetDE). We employed a REGEX string to identify each repeat, defined as in Hu *et al*. [39], and used the aa at positions 10, 11, 12, 14, 30, 32 and 39 for classification based on their high dN/dS ratios [30].

Pairwise physicochemical dissimilarities between the different repeat variants were calculated based on the same seven high dN/dS positions by summing the pairwise distances at each position, as given by the aa physicochemical dissimilarity matrix in [60]. To generate colour palettes for displaying the repeats, colours were chosen in the 3D CIE L*a*b* colour space [61] using a variant of non-metric multidimensional scaling, which matches the relative physicochemical distances of the repeat variants as closely as possible to the relative perceptual distances of the colours, while constraining the output to colours, which can be represented in the sRGB gamut. This palette generation algorithm was inspired by Gecos [62] and was implemented as a custom R script (available at https://github.com/SLAment/FixingHetDE) using the Python source for Gecos (https://github.com/biotite-dev/gecos) as reference.

To clarify the relationships between *het-d* and *het-e*, we extracted the sequences of all NWD genes containing HIC WD40 repeats from the reference genome S+ (except for *nwd6*, in which case we used the sequence from strain Wa21-). We produced a general protein alignment with the online version of mafft 7 [63] using default parameters (BLOSUM62 scoring matrix, gap penalty of 1.53). We then divided the alignment into four sections: the HET domain (positions 1–293 of the HET-E protein, GenBank accession number CDP27944.1) when present and the NACHT domain and the start of the WD40 domain (positions 294–978) split into three sections of roughly the same size. Preliminary analyses showed high levels of long-branch attraction in the NACHT subphylogenies. Hence, we removed ambiguous sites from the alignments with trimAl v1.4.1 [64] and the -strictplus option. We used IQ-TREE v2.2.3 [65] with automatic selection of substitution model (-m MFP) and 100 bootstrap pseudo-replicates (-b 100) to produce maximum likelihood phylogenies. Trees were visualized with FigTree v1.4.4 (https://github.com/rambaut/figtree).

To compare the WD40 repeats of all NWD genes, we produced an aa sequence logo per gene with the R package ggseqlogo v0.2 [66]. The nt sequences of the WD40 repeats were read into R with the ape package v5.7 [67]. We produced principal component analyses (PCAs) with the packages adegenet v2.1.10 and ade2 v1.7-22 [68].

### Cloning and transformation of *het-e*

Our isolate of Wa63+ had poor growth, and subsequent DNA extractions were done from its sibling Wa63−, which is largely isogenic [69] and has an identical *het-e* allele (confirmed by PCR). The *het-e* Wa63- gene was PCR amplified with oligonucleotides osd192 (CAAGGTTGTGGCGGTTTCAG) and osd193 (GCGTTTGACAAGACGGTGAC) (respectively positioned at 605 nt upstream and 459 nt downstream of the ORF) on 33 ng of genomic DNA extracted from the Wa63- strain using the Q5^®^ High-Fidelity 2X Master Mix (New England Biolabs, M0492S). We cloned 50 ng of PCR product in a pCR-Blunt II-TOPO^®^ vector using a Zero Blunt TOPO^®^ PCR Cloning Kit (Invitrogen, 45-0245) according to the manufacturer’s protocol. The ligation reaction was diluted at 1:4 in water, and chemically, NEB^®^ 5-alpha Competent *E. coli* (New England Biolabs, C2987H) were transformed with a twelfth of the ligation reaction.

DNA transformation was performed as previously described [70] using the p1 vector, a pBlueScript-II derived vector containing the *nat1* nourseothricin acetyltransferase gene in co-transformation and using 5 µg of the *het-e*-bearing plasmid and 1 µg of the p1 co-transformation vector. The recipient strains for transformation were *C2d3e4* (*het-c2 het-d3 het-e4*) and *C1d3e4* (*het-c1 het-d3 het-e4*). Five days after transformation, 24 individual transformants in each transformation were tested in barrage assays against the 4 common *het-c* alleles on corn meal agar. The tester and recipient strains were created by backcrossing known alleles from the French strains into the s strain background [38]. Specifically, the *E1* and *E2* strains were derived from the strains A and C, respectively. Likewise, the *C1*, *C2*, *C3* and *C4* strains were derived from the strains A, s, H and M [38].

The *D1^A^* allele was cloned and transformed with the same method, but using the oligonucleotides CCTCCTTCAGCGTAGTCGAC and TGAGCAGCGTGTACTGAAGG on DNA extracted from the CaDa+ strain.

### Prediction of protein structure

We used the server of AlphaFold 3 [71] available at https://alphafoldserver.com/ to model the protein structure of the WD40 domain. We used the protein sequence of the second HIC repeat in the *E2*^C^ allele (TGTQTLEGHGGSVWSVAFSPDGQRVASGSDDKTIKIWDAASG) as an arbitrary representative of a typical *het-e* HIC repeat. We gave this repeat to AlphaFold in 6, 7, 8 and 9 copies (multimers) to assess their predicted structure with default parameters. In addition, we input the protein sequence of HET-C2 (GenBank accession number AAA20542.1) and HET-E1^H^, also with default parameters. Only the first predicted model (number 0) was considered. We visualized protein structures in USCF ChimeraX v1.8 [72].

## Results

### The haplotypes of HNWD genes can be recovered consistently from long-read data but not from short-read assemblies

To assess the consistency of HNWD genes across sequencing efforts, we first focused on three *P. anserina* strains: the Dutch strain Wa63+ and the French strains Y+ and Z+ (the + and - annotations signify the mating type). These three strains are haploid, and their genomes were originally sequenced as paired-end (125 bp×2, insert size ~350) libraries with Illumina HiSeq 2500 at high coverage (>80×) [43]. In the same study, HMW DNA was also extracted from Wa63+ and Y+, which was then sequenced using either PacBio RSII or an R9 ONT flowcell, respectively [43]. Here, we resequenced these same strains using R10 ONT flowcells in a barcoded library (see Methods). Hence, this dataset allowed us to compare the HNWD haplotypes obtained from different sequencing technologies and assemblies of the same strains at different time points (Tables 1 and S1, available in the online Supplementary Material).

**Table 1.** Whole-genome assemblies of *P. anserina* strains used in this study

Strain*	Origin	Sequencing technology	Assembler	Mean depth (×)	Mean read length (bp)	Scaffolds†	Source of sequencing data
Wa63+	NL	PacBio RSII	HGAP 3.0	111.22	12,562	7	Vogan et al. [43]
		ONT R10	Flye 2.9.3	79.26	3,268	9	This study
		HiSeq 2500	SPades 4.0.0 21, 33, 55, 77 k-mers	88.87	125	3,141	Vogan et al. [43]
		HiSeq 2500	SPades 4.0.0 default k-mers	89.00	125	1041	Vogan et al. [43]
Y+	F	ONT R9	Miniasm 0.2	83.72	1,952	8	Vogan et al. [43]
		ONT R10	Flye 2.9.3	107.62	2,685	9	This study
		HiSeq 2500	SPades 4.0.0 21, 33, 55, 77 k-mers	97.67	125	2,746	Vogan et al. [43]
		HiSeq 2500	SPades 4.0.0 default k-mers	97.51	125	926	Vogan et al. [43]
Z+	F	ONT R10	Flye 2.9.3	80.94	2,050	12	This study
		HiSeq 2500	SPades 4.0.0 21, 33, 55, 77 k-mers	97.24	125	2,824	Vogan et al. [43]
		HiSeq 2500	SPades 4.0.0 default k-mers	97.13	125	1,041	Vogan et al. [43]
Wa137−	NL	ONT R9	Miniasm 0.2	49.92	7,913	8	Vogan et al. [44]
		HiSeq X	SPades 4.0.0 21, 33, 55, 77 k-mers	91.31	150	8,321	Vogan et al. [44]
Wa21−	NL	PacBio RSII	HGAP 3.0	80.30	11,863	9	Vogan et al. [43]
Wa28−	NL	PacBio RSII	HGAP 3.0	76.20	10,105	7	Vogan et al. [43]
Wa46+	NL	PacBio RSII	HGAP 3.0	117.82	12,949	9	Vogan et al. [43]
Wa53−	NL	PacBio RSII	HGAP 3.0	83.82	11,382	7	Vogan et al. [43]
Wa58−	NL	PacBio RSII	HGAP 3.0	112.17	13,130	7	Vogan et al. [43]
Wa87+	NL	PacBio RSII	HGAP 3.0	105.87	12,928	9	Vogan et al. [43]
Wa100+	NL	PacBio RSII	HGAP 3.0	114.09	12,857	7	Vogan et al. [43]
T_G_+	F?	ONT R9	Miniasm 0.2	37.67	1,384	13	Vogan et al. [43]
S+	F	Sanger	Arachne	−	−	7	Espagne et al. [53]
CmEm−	Lab	ONT R10	Flye 2.9.3	24.04	6,831.2	7	This study
CoEc+	Lab	ONT R10	Flye 2.9.3	68.98	3,856.2	9	This study
CoEf+	Lab	ONT R10	Flye 2.9.3	66.13	4,780.7	9	This study
ChEhDa+	Lab	ONT R10	Flye 2.9.3	110.49	4,560	10	This study
CaDa−	Lab	ONT R10	Flye 2.9.3	67.39	4,275.3	11	This study
CsDf+	Lab	ONT R10	Flye 2.9.3	38.90	4,670.4	9	This study

*The + and − signs refer to the strain’s mating type.

†The number of scaffolds for long-read data corresponds to those that map to the seven chromosomes (excluding mitochondrial and rDNA contigs), but all scaffolds for the short-read datasets.

NL, Wageningen, the Netherlands; F, France; Lab, lab strain.

**Fig. 2.**
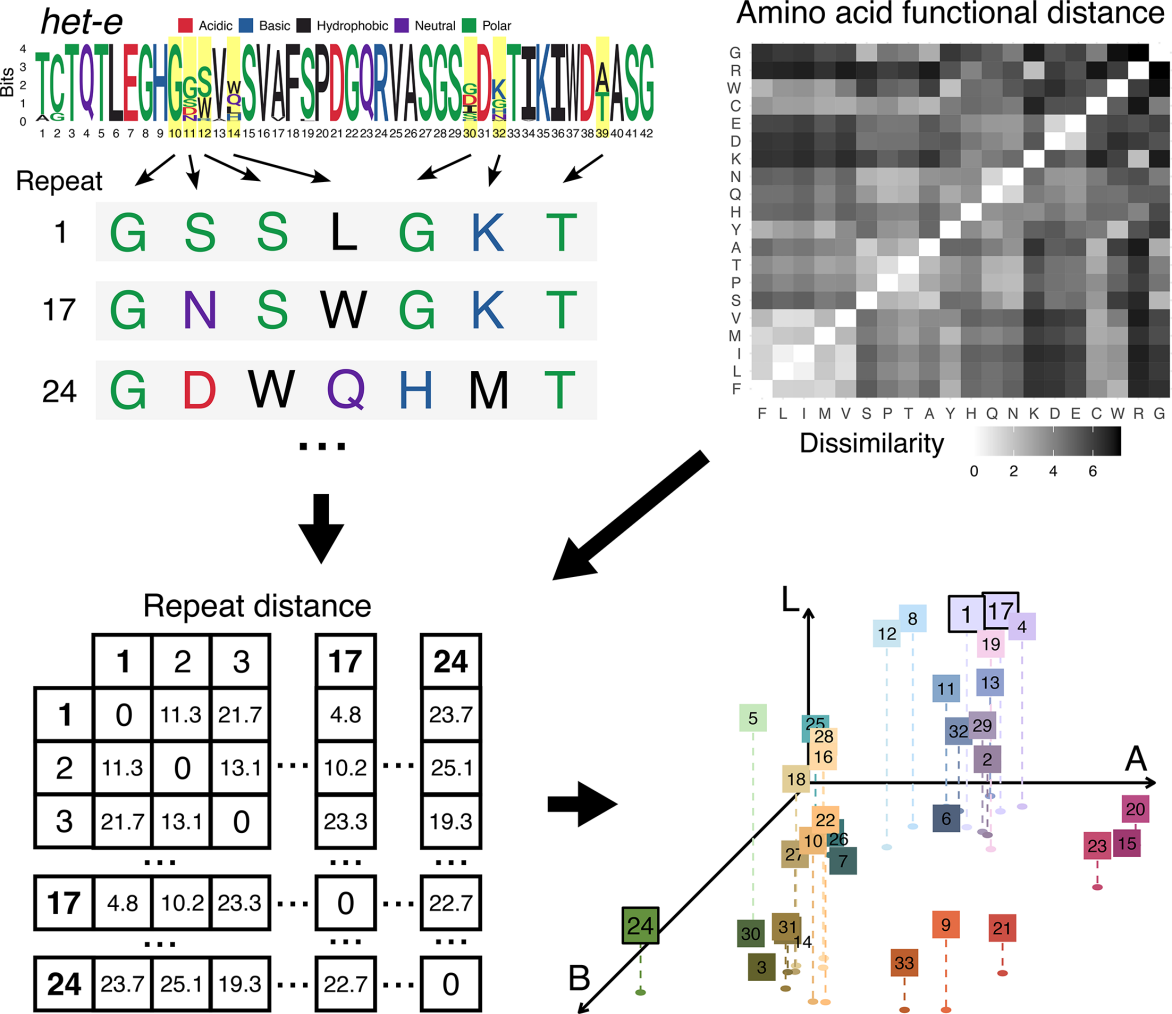
Colour classification of WD40 repeats with HIC using *het-e* as an example. The individual repeats were categorized based on the unique combination of aa present at seven positions, highlighted in a logo diagram of *het-e* on the top left corner. A matrix of functional dissimilarity of aa (upper right) was then used to calculate pairwise distances between repeat variants (lower left), where the dissimilarity of two variants is the sum of the dissimilarities of their aa at each of the seven selected positions. These distances were then used to project the repeat variants into the 3D L*a*b colour space, such that repeats with functionally similar aa are assigned perceptually similar colours (lower right). Each repeat variant is plotted in L*a*b space as a coloured square, along with a dashed line connecting it to a point at its projection in the a*b plane. Three repeats are highlighted to illustrate the case of one distant and two similar repeats (1, 17 and 24).

We extracted the sequence of *het-d*, *het-e* and *het-r* from each assembly and compared their WD40 domain (Figs2  3). To facilitate the visualization, we classified the HIC repeats of each gene by assigning an arbitrary number based on unique combinations of seven aa at positions previously inferred to be under diversifying selection [30] (Table S2). These numbers were only used as identifiers and hold no other meaning. We then calculated dissimilarity scores among the HIC repeat classes based on an aa physicochemical dissimilarity matrix [60]. The scores were used to generate palettes in the CIE L*a*b* colour space, such that similar colours imply similar physicochemical characteristics (Fig. 2). We found that repeats were more different between paralogues than among the repeats of each paralogue (see below), so we assigned an independent palette per gene to facilitate contrast. See also Figs S1, S2 and S3.

**Fig. 3.**
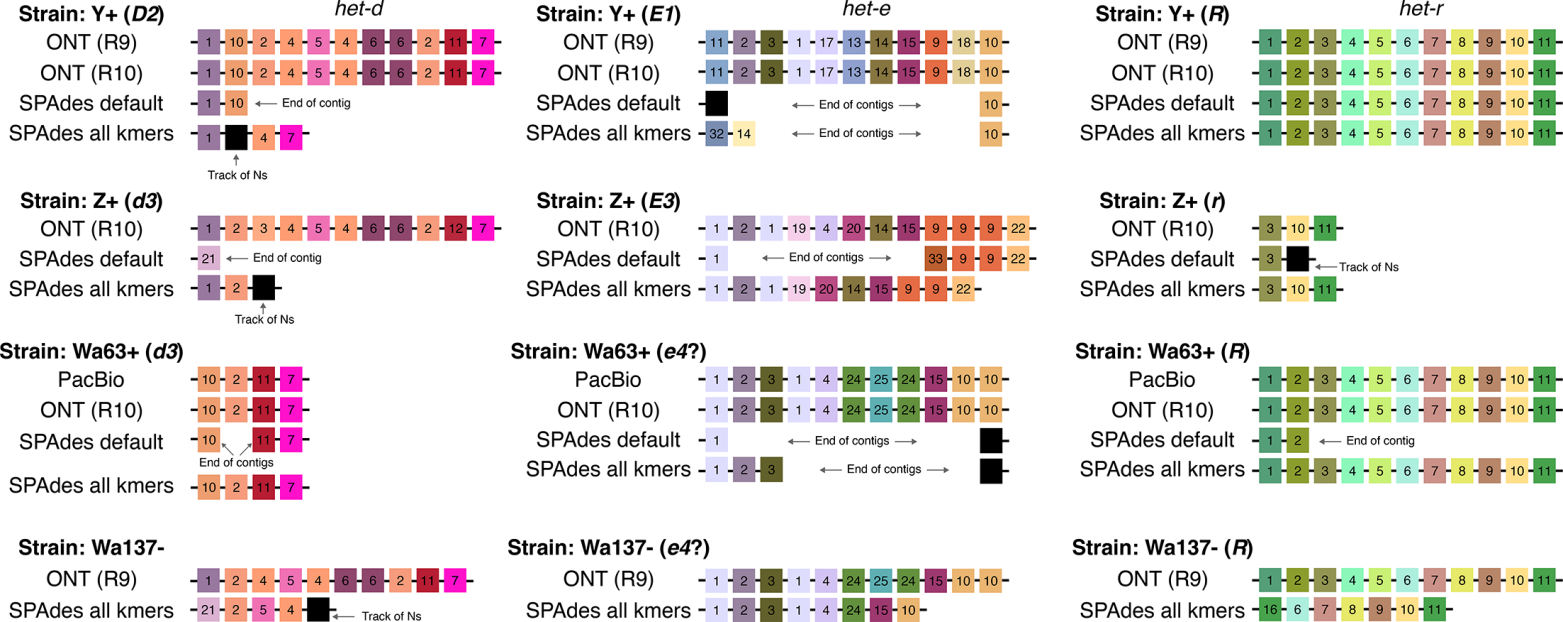
Assembly of the WD40 domain from different sequencing technologies. Only repeats with HIC are shown. Each repeat was arbitrarily classified based on unique aa combinations (see Table S2), but the colours reflect their physicochemical similarity (each gene has an independent palette). Repeats with a track of missing data (Ns) are coloured black. Black lines linking the repeats symbolize the containing scaffold.

We found that the long-read assemblies were in agreement, regardless of the technology and assembler (Fig. 3). In the case of *het-r*, both Y+ and Wa63+ recovered the exact same sequence of repeats as reported for the reference *R* allele [35]. This suggests that (1) the sequence obtained from the long reads (and the reference itself) is correct and (2) the HNWD alleles did not mutate despite an unknown amount of vegetative growth and culture transfers that the strains have undergone in the lab since isolation.

Most sequencing datasets of non-model fungal species are based on short-read data, implying that NLRs with HIC are usually assembled from Illumina reads. What is the likelihood that these assemblies are correct? As a proof of concept, we used the popular short-read assembler SPAdes [57] to test if we can obtain equivalent haplotypes from the published Illumina data of Wa63+, Y+ and Z+. SPAdes constructs assembly graphs using multiple k-mer sizes, which can be selected automatically by the programme [57]. In our case, the selected k-mer sizes were 21, 33 and 55 bp (‘default’ treatment). To promote contiguity in the assembly, we also produced assemblies using an additional larger k-mer of 77 bp (the ‘all k-mers’ treatment). As before, we extracted the WD40 domain of the HNWD genes but found that it is often fragmented into two scaffolds (Fig. 3). In cases where an HNWD gene was fully contained within a scaffold, the haplotypes harboured tracks of missing data (Ns) or presented a different sequence than their long-read data counterparts (e.g. *het-e* in the strain Z+). The only exceptions where long (>5 repeats) alleles were correctly recovered from Illumina assemblies were those of *het-r* in the ‘all k-mers’ SPAdes assemblies (Fig. 3). Notably, some SPAdes assemblies included chimeric repeats that were not present in the WT long-read haplotypes (e.g. repeat variant 32 in the ‘all k-mers’ *het-e* sequence of the strain Y+).

The Illumina reads of Wa63+, Y+ and Z+ are relatively short, of 125 bp. Current Illumina technologies usually have slightly longer reads of 150 bp. To assess if that difference was enough to recover the HNWD alleles, we used published data of the strain Wa137- [44]. The genome of this strain was sequenced with R9 ONT flowcells as for Y+, but its short-read library was sequenced with the HiSeq X machine (150 bp paired-end, insert size ~250 bp) (Table 1). For this read length, SPAdes defaults to all k-mers (21, 33, 55 and 77); hence, we only evaluated the assembly with those parameters. As with the other strains, we found that the HNWD haplotypes recovered are shorter than the long-read assembly, omitting or creating repeats (Fig. 3). We conclude from these analyses that HIC repeats are not recovered confidently from Illumina assemblies, which calls for caution when analysing HIC NLRs in published short-read genomes.

### Different WD40 repeat combinations result in the same functional allele

The molecular biology studies that first described *het-d* and *het-e* used lab strains constructed by backcrossing the alleles of French strains with known phenotypes into the genomic background of a reference strain (s, also referred to as ‘little s’) [32,33, 35, 36]. We sequenced the genome of some of these backcrossed strains using ONT R10 as above. The backcrossed strains are designated by their reactive genotypes. For example, the strain CmEm- contains the *het-c* (*C2*^M^) and *het-e* (*E2*^M^) alleles of the French strain M, while having the non-reactive *het-d* allele (*d3*^s^) of strain s. Likewise, the strain ChEhDa+ has the *het-c* (*C3*^H^) and *het-e* (*E1*^H^) alleles of the H strain and the *het-d* allele (*D1*^A^) of the A strain. The exceptions are strains with a null *het-c* allele, here termed Co (CoEc+ and CoEf+). The genome assemblies of these backcrossed strains consist of mostly full chromosomes or chromosome arms (Table S1).

**Fig. 4.**
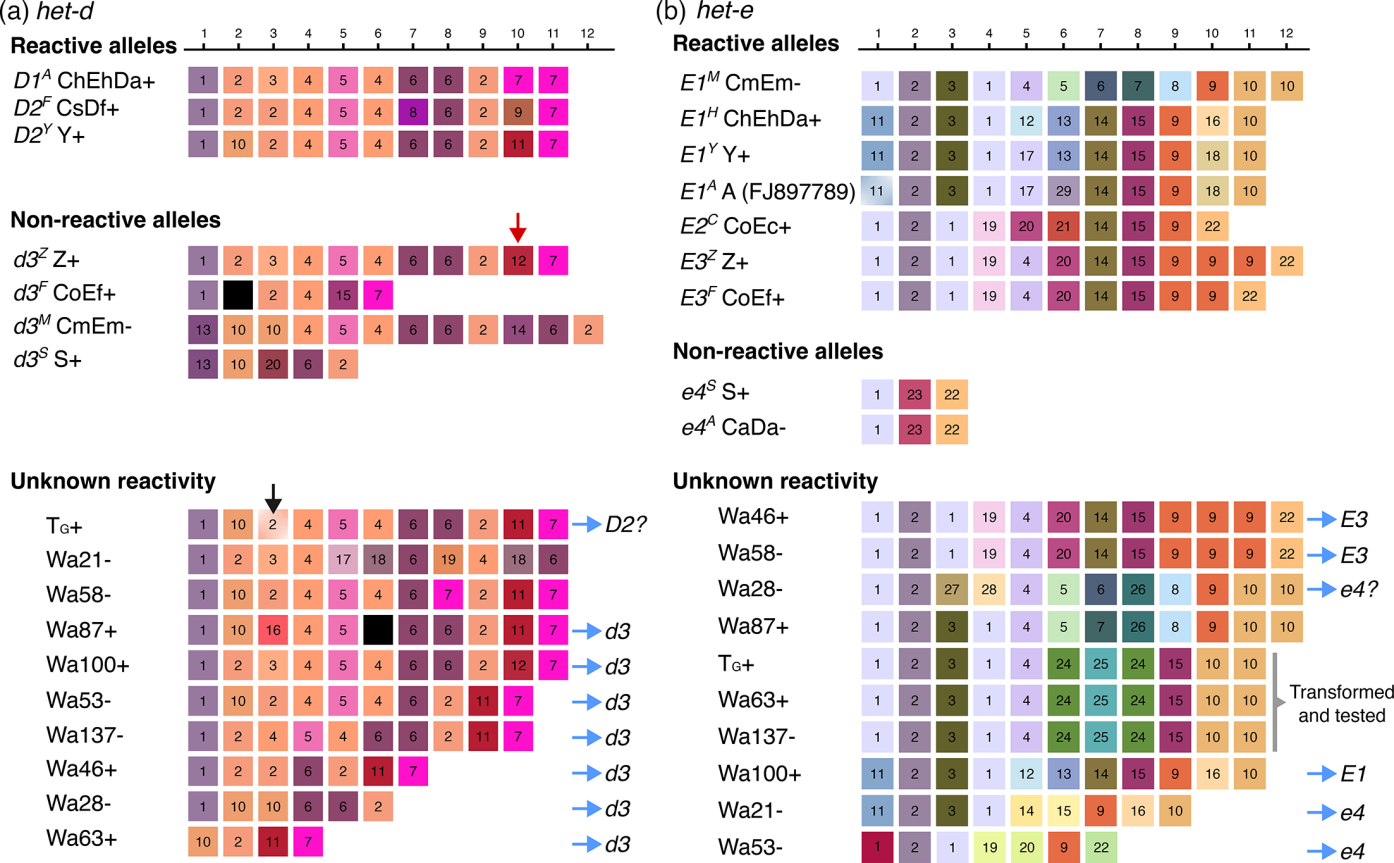
Long-read assemblies of *het-d* (**a**) and *het-e* (**b**) WD40 domain from different WT strains. Only repeats with HIC are shown, arbitrarily classified based on unique aa combinations (see Table S2), but coloured based on their physicochemical similarity (each gene has an independent palette). Repeats containing stop codons or frameshifts are coloured black. The red arrow highlights the single repeat distinguishing the reactive *D1^A^* allele (in strain ChEhDa+) from the non-reactive *d3^Z^* allele (Z+). The black arrow marks the repeat with a deletion in the T_G_+ sequence that is likely a misassembly. Blue arrows point to inferred alleles based on sequence or the number of repeats. A specific allele of *het-e* was selected for phenotypic testing. The beginning of the first repeat is missing in the *E1*^A^ sequence (GenBank accession number FJ897789), but the missing aa happen to be perfectly conserved in all sequences, and hence, we inferred it to be identical to the first repeat of *E1*^Y^.

The *het-e* and *het-d* sequences from these new genomes add to the collection of reliable sequences for alleles of known reactivity. The ChEhDa+ and CaDa- strains have the same *het-d* allele as the A strain (*D1*^A^), and the recovered sequences were identical, reinforcing the notion that long-read assemblies represent the real DNA sequence. The strain Z+ above belongs to the original collection of French strains with known phenotypes [28]. The strain S+ (‘big S’) is also part of this collection, and its genome is considered the reference for the species, although it predates long-read technologies [53]. However, S+ has non-reactive *het-d* and *het-e* alleles that are relatively short (e.g. Fig. 1c) and hence more likely to be correctly assembled. In addition, the strain Y+ is known to harbour a *D2* allele [33] and an *E1* allele (L. Belcour, personal communication). Hence, current data allows preliminary comparisons of intra-allele variation (Fig. 4).

In agreement with previous reports, our long-read assemblies show that all the reactive *het-d* alleles have 11 repeats with HIC, while the reactive *het-e* alleles can be 10, 11 or 12 repeats long (Fig. 4). However, the precise order and identity of the repeats from the published *D2*^Y^ and *E2*^C^ alleles show strong differences with the ONT assemblies (Fig. S4). Likely, older methodologies had difficulties establishing the specific order of repeats, but the general inference that the *het-d* and *het-e* alleles with less than ten repeats are non-reactive still holds [32,33]. Indeed, many of the non-reactive sequenced alleles are short (Fig. 4). Nonetheless, as pointed out previously [32,33], having the right number of repeats is not enough to create a reactive allele. A clear example is given by the non-reactive *het-d* allele of strain Z+ (*d3*^Z^), which is identical to the *D1*^A^ allele except for a single repeat at the tenth position (red arrow in Fig. 4a). This repeat differs from its functional counterpart by just four aa (Table S2) and has a single aa difference with the repeat variant 11 at the same position in *D2*^Y^. The fact that some repeat positions are highly conserved across allele classes further suggests that these positions are key for functionality (e.g. the fourth to sixth positions of *het-d* and the second position of *het-e*).

Interestingly, none of the sequences from the same allele class are identical (e.g. all the different *E1* alleles), implying that although a single misplaced repeat can break an allele, there must be some flexibility at some repeat positions. For example, there is considerable intra- and inter-allele variation at the fifth and sixth repeat positions of *het-e* (Fig. 4b). The *E1*^M^ allele (CmEm-) is particularly puzzling since it is quite different from the other *E1* alleles at the fifth to ninth repeats. Nonetheless, some positions might be diagnostic of an allele class. For example, all the *E1* alleles have the same repeat at the third and fourth positions, as well as the same last repeat (regardless of the haplotype length), relative to the *E2* and *E3* alleles. Notably, the length polymorphism seems to be concentrated towards the last three repeats of the *E* alleles, with the *E3* alleles being the best illustration. In this case, a single repeat (classified as variant 9 in Fig. 4b) is repeated in *E3*^Z^ relative to *E3*^F^. Likewise, the *E1*^M^ allele has an extra variant 10 repeat compared with the other *E1* alleles.

Having established a reference panel of allele sequences, we looked at published long-read data of other WT strains [43,44]. While T_G_+ and Wa137- were sequenced with ONT R9, the other strains were sequenced using the PacBio RSII technology [43,44]. From these strains, Wa100+ has the same *het-d* sequence as Z+ and hence has a *d3* allele (Fig. 4a). The *het-d* sequence of T_G_+ is in fact very similar to that of *D2*^Y^, with the notable exception of a single bp deletion at the third repeat (Fig. 4a) and a substitution in position 17 (not under diversifying selection) of two repeats. Inspection of the short-read mapping to the assembly of this strain suggests that the deletion is a misassembly within a small homopolymer track (Fig. S5), which is a more acute problem in R9 data than R10 or PacBio [41]. On the other hand, the strain Wa87+ has two stop codons in its sixth repeat that are supported by read mapping (Fig. S6). Hence, this strain likely has a disrupted protein and can be tentatively assigned to a *d3* allele type. All strains with less or more than 11 repeats can also be considered *d3* [33].

In the case of *het-e*, three strains have sequences identical to those in the reference panel: both Wa46+ and Wa58- have an *E3* allele, while Wa100+ has an *E1* allele (Fig. 4b). Based on the similarity to *E1*^M^, the sequence of Wa87- might be *E1*, although that requires testing. The sequence of Wa28- also resembles *E1*^M^ but has two very different repeats at the third and fourth positions. This strain contains a very rare *het-c* allele known as *C8*, capable of recognizing all three reactive *het-e* alleles [73]. Thus, Wa28- likely has an *e4* allele. Lastly, sequences with less than ten repeats can be assigned to the *e4* allele.

### The *het-e* allele of Wa63+ does not recognize common *het-c* alleles

**Fig. 5.**
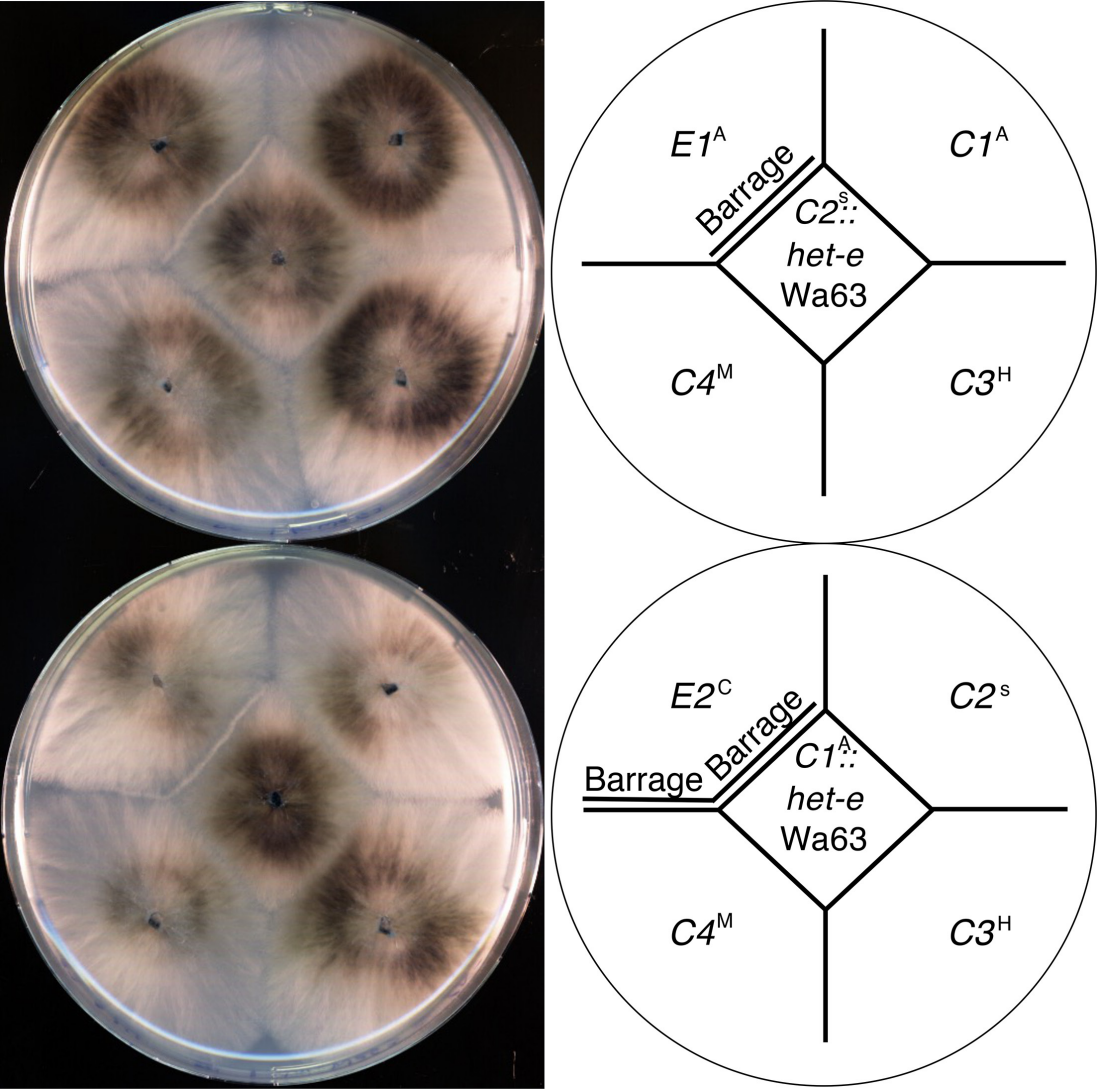
The *het-e* allele from Wa63 is compatible with the four common *het-c* alleles. Barrage assay of *C1* and *C2* strains transformed with the *het-e* allele of Wa63 cloned on a plasmid and tested with the four common *het-c* alleles. The *C2* recipient strain (upper plate) allows for testing against *C1*, *C3* and *C4*. The *C1* recipient (bottom plate) allows for testing against *C2*, *C3* and *C4*. The *E1* and *E2* alleles (on the upper left on the upper and bottom plates, respectively) are used for positive controls for the barrage (incompatibility) reaction. Note the barrage formation between the *E2* and *C4* testers in the bottom plate. In the strain designation, the *het-c*, *het-d* and *het-e* genotypes are omitted for clarity when strains carry inactive alleles. In other words, a *C1* strain has *d3* and *e4* alleles, an *E1* strain has a *d3* and a null *het-c* allele, etc. The source strain of each active allele is marked as a superscript (e.g. *E1^A^* indicates that *E1* comes from the A strain).

Although this is a small sample of strains, we noticed that one particular *het-e* sequence appeared in three WT strains: T_G_+, Wa63+ and Wa137- (Fig. 4b). The origin of T_G_+ is unclear, although it might correspond to the French T strain [43]. The other two strains were both sampled in Wageningen, the Netherlands, but one in 1994 and the other in 2016. Hence, we wondered if this was an unidentified functional *E* allele. As these strains have *C2*, *C9* and *C2* alleles, respectively, then their *het-e* sequence cannot correspond to *E1* or *E2* alleles, as that would create self-incompatibility. To assess its reactivity, we cloned the *het-e* allele of Wa63- (identical to Wa63+) on a plasmid and introduced it by transformation into two different recipient lab strains with no reactive *het-e* or *het-d* alleles: one with a *C1d3e4* genotype and another with a *C2d3e4* genotype (Fig. 5). In this way, it is possible to assay incompatibility to the common *het-c* alleles (*C1*, *C2*, *C3* and *C4*) using previously produced lab strains [38]. In total, 24 transformants were tested in barrage assays against the testers carrying the common *het-c* alleles. We found that all transformants were compatible with all *het-c* testers. In a control experiment, using a cloned *D1^A^* allele introduced into the *C1d3e4* recipient, 15 out of 24 tested transformants produced a barrage reaction. We conclude from this experiment that the *het-e* allele from Wa63+ does not lead to incompatibility with the common *het-c* alleles. Either this allele is inactive in incompatibility or, alternatively, it could lead to incompatibility to rare *het-c* alleles that were not tested in this experiment (see Discussion). Notably, this allele lacks a repeat variant 9, which is present towards the end of all known functional alleles.

### The reactive HNWD alleles likely form a double *β*-propeller structure with cryptic repeats

The WD40 *β*-propeller fold is formed by six to eight, but usually seven, units called ‘blades’, which are arranged radially around a central tunnel [31]. Each blade, in turn, is formed by four antiparallel *β*-sheets named *a*, *b*, *c* and *d*. By convention, a WD40 repeat does not exactly correspond to a blade, but instead starts with a *d β*-sheet from the previous blade, followed by *a*, *b* and *c* sheets of the focal blade (Fig. S7A). To close the circle, the last blade is often constructed from one to three *β*-sheets of the last repeat (the C-terminus), complemented by remaining *β*-sheets from the N-terminus, a configuration known as the molecular ‘velcro’ [31,74].

Our results confirm that the most common reactive HNWD alleles display 11 HIC repeats, which would represent an atypical blade number. We turned to AlphaFold 3 to model the WD40 *β*-propellers of *het-e*. In the first experiment, we input a single repeat (the second HIC repeat in the *E2*^C^ allele) and modelled different combinations of blade numbers, from 6- to 9-mers. The AlphaFold model quality and confidence scores (pTM and ipTM, where 1 represents the best prediction) were highest for 7-mers and 8-mers (Fig. S7A). As an alternative approach, we created an artificial sequence of 6, 7, 8 and 9 tandem identical repeats (Fig. S7B). In this case, the 7-repeat sequence yielded the highest pTM value (0.95), suggesting seven is a relevant size for the *het-e β*-propellers.

**Fig. 6.**
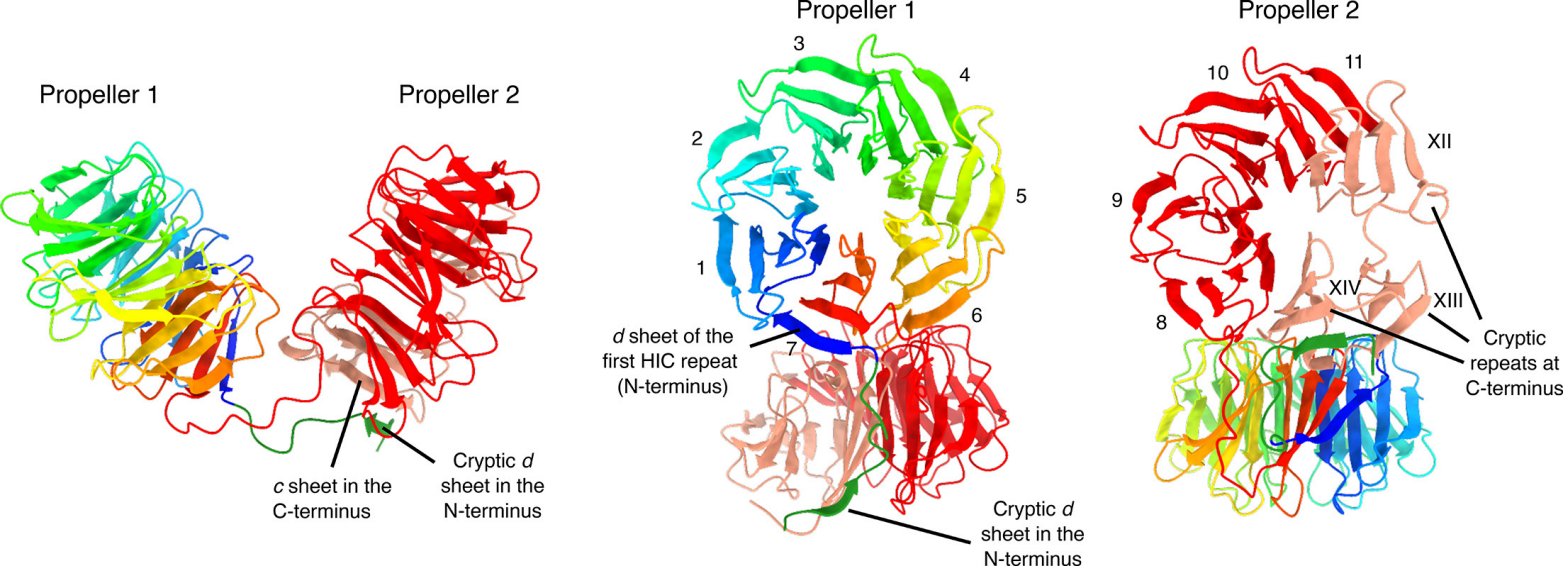
Ribbon diagrams of the WD40-domain structure from the HET-E1 protein (*E1*^H^ allele) produced by AlphaFold 3. The first 817 sites containing the HET and NACHT domains were removed for clarity (pTM=0.69 for the full model). The first propeller is coloured with a rainbow palette to illustrate the direction of the individual *β*-sheets. The cryptic *d β*-sheet in the N-terminus of the WD40 domain that forms the molecular velcro with the C-terminus is also highlighted (forest green). The second propeller is coloured based on HIC (red) and cryptic (salmon) repeats. Individual blades are numbered with Latin (HIC) or Roman (cryptic) numerals.

Next, we modelled the HET-E1^H^ allele as a representative reactive HET-E protein (Fig. 6). In the context of the full-length protein, the WD40 domain is modelled as two independent 7-mer *β*-propellers. Importantly, the second predicted propeller is made of four canonical highly conserved blades (eighth to eleventh) and three additional cryptic (divergent) blades located at the C-terminus of the protein, closed by a molecular velcro with a cryptic *d β*-sheet on the N-terminus (Fig. 6). These modelling approaches suggest that active WD40 repeat domains have a mosaic structure with two propellers forming a clamp-like shape, one of which comprises a combination of four canonical and three cryptic repeats. Such a model provides a plausible explanation for the occurrence of an otherwise unusual number of repeats in active *het-d/e* alleles.

We also modelled a tentative HET-C2/HET-E1^H^ protein complex (Fig. S8A and B). Although with relatively weak support scores (ipTM=0.43 and pTM 0.60), the resulting model is consistent with previous observations. AlphaFold 3 places the HET-C2 protein, whose model is fully congruent with the experimental HET-C2 X-ray structure (PDB 3KV0; Fig. S8C), within the clamp-like assembly formed by the two propellers (Fig. S8A and D). The HET-C2 protein is positioned at the predicted interaction surface that contains the WD40 hypervariable sites under diversifying selection [30]. Similarly, the HET-C residues known to define allele specificity or that are under positive selection [73] are also located in the predicted interaction surface, with the exception of residue 118 (Fig. S8D). At this position, the rare allele *C8* has a replacement of cysteine by a bulky and positively charged arginine residue [73], an alteration that, despite not being directly at the interaction surface, may be drastic enough to affect specificity.

### The *het-d* and *het-e* genes evolved the capacity to recognize *het-c* independently despite repeat exchange between NLRs

Given their similarities, it is unclear if *het-d* and *het-e* are able to recognize the same ligand (*het-c*) because of common ancestry or convergent evolution. Along with *het-r*, the HNWD gene family contains two other members of unknown function, *hnwd1* and *hnwd3*. The HNWD genes in turn are part of a larger group of NLRs known as the NWD genes, which also have NACHT and WD40 domains, but their effector domains are different from HET [30]. The NWD genes include at least two pseudogenes, *nwdp-1* and *nwdp-2*, based on the reference genome of the strain S+. Upon closer inspection in our data, we noted that *nwdp-1* appears to be functional (i.e. without stop codon mutations) in some strains. Hence, here, we renamed *nwdp-1* as *nwd6* to emphasize the fact that it might be functional depending on the strain.

**Fig. 7.**
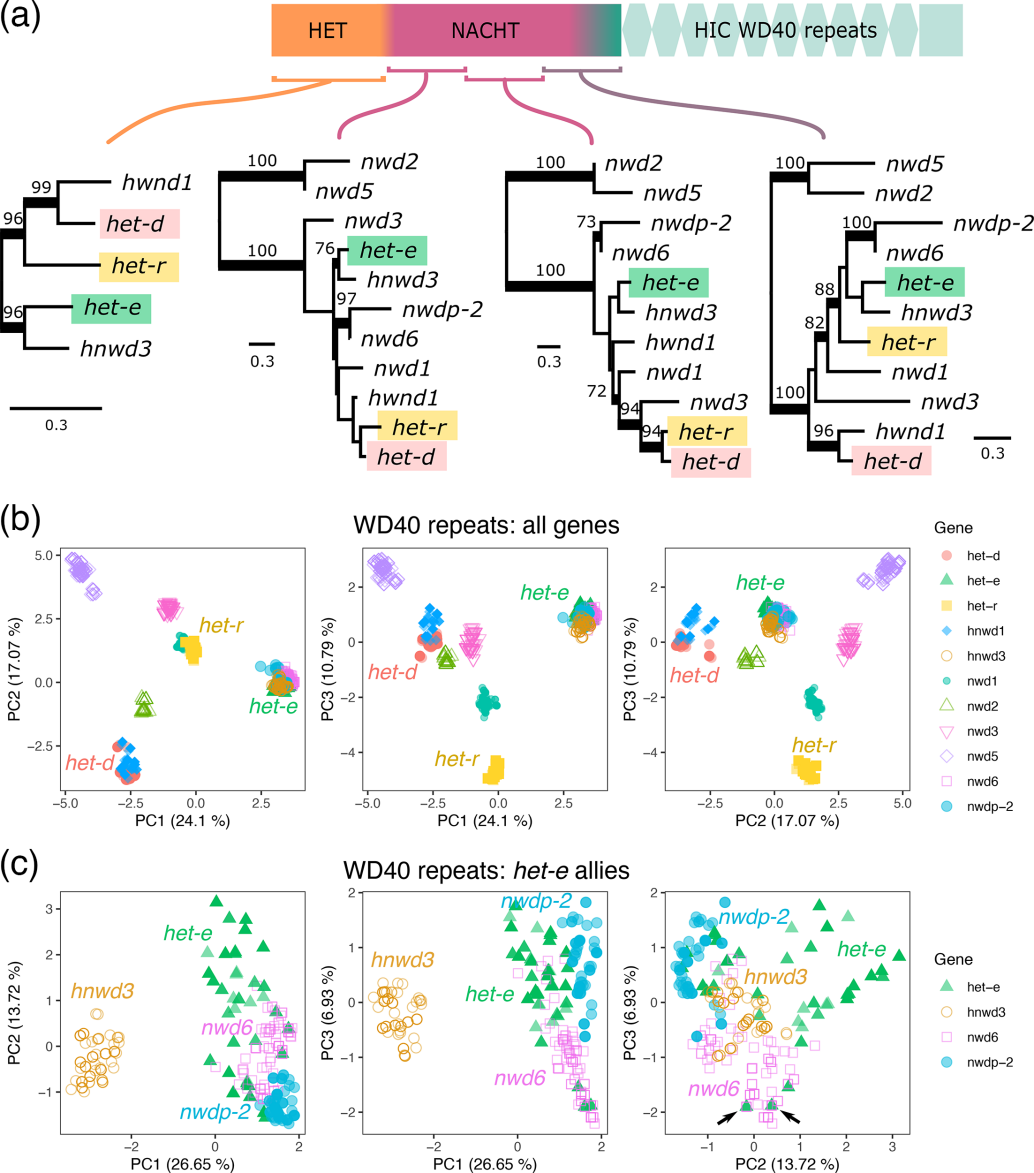
Relationship between different domains of HNWD genes and related NLRs. (**a**) Maximum likelihood phylogenies along the HET, NACHT and WD40 domains. Branch support values correspond to standard non-parametric bootstrap (values <70 omitted). Branches are proportional to the scale bar (aa substitutions per site). The beginning of the WD40 domain contains a cryptic (divergent) repeat, used to make a phylogeny, followed by repeats with HIC. (**b**) The individual nt sequences of the HIC WD40 repeats were extracted from all *P. anserina* long-read assemblies and analysed in a PCA. (**c**) PCA of HIC WD40 repeats in genes closely related to *het-e*. The two repeats identical between *het-e* and *nwd6* are easiest to see when comparing the principal components 2 and 3 (arrows).

To evaluate the relationship between *het-d* and *het-e*, we performed phylogenetic analyses on the NWD genes (Fig. 7a). We used the sequence of the reference strain S+ for most genes, with the exception of *nwd6*, which was extracted from the Wa21- assembly. Notably, we found that genealogies of different gene parts display slightly conflicting relationships: the HET domain and the beginning of the WD40 domain (containing part of the cryptic repeats) suggest that *hnwd1* is the closest relative of *het-d*, while phylogenies of most of the NACHT domain recover *het-r* as the closest relative of *het-d*. Regardless, all trees show that *het-d* and *het-e* are not as closely related. Interestingly, a broader phylogenetic analysis on the HET+NACHT domains including all alleles in our dataset revealed a phylogenetic structure within the *het-e* clade that partially tracks the functional alleles (Fig. S9).

As the binding specificity of the NWD genes depends on the HIC repeats, next we extracted the nt sequence of all individual WD40 repeats in the long-read *P. anserina* assemblies, amassing a total of 1,107 WD40 repeats. We performed a PCA on the nt alignment and found that the repeats from most genes are strongly differentiated (Figs 7b and S10). Importantly, the repeats from *het-d* and *het-e* can be found on very different sides of the variation space.

Although weakly supported, the phylogenetic analyses suggest that *hnwd3* is the closest relative of *het-e* (Fig. 7a). We found that while *hnwd3* repeats are clearly distinct, the repeat variation found in *het-e* overlaps with that of *nwd6* and its pseudogenized paralogue *nwdp-2* (Fig. 7c). In fact, we found two repeats that have an identical nt sequence between *het-e* and *nwd6*, which is remarkable given the high divergence at the protein level of the other domains. Hence, we interpret these observations as evidence of repeat exchange between genes, as suspected previously [30]. However, there is no indication of exchange between *het-d* and *het-e*. Indeed, the *het-d* repeats are much closer to those of *hnwd1* (Figs 7b and S12). Hence, we conclude that *het-d* and *het-e* independently evolved the capacity to recognize *het-c* and trigger an allorecognition reaction.

## Discussion

Evolutionary and functional research of immune system genes, including NLRs, often comes with technical and methodological challenges. For example, rapid evolution might limit phylogenetic reconstructions or ascertainment of homology [75]. Likewise, the presence of paralogues and association with transposable elements can result in fragmented genome assemblies at precisely the NLR locations [40,76]. Thus, highly similar repeats at the C-terminal domain (i.e. HIC) can act as the final nail in the coffin for the assembly of certain NLRs. Here, we show that current long-read technologies fully overcome that problem for the WD40 domain of HNWD genes, regardless of the software and the technology used. By sequencing the alleles used in classical genetic studies, we provide confidence to older inferences on the characteristics of reactive alleles but highlight the potential inconsistencies of Illumina-derived assemblies in general. These high-confidence sequences, in turn, can be used to infer the phenotype of other strains, bypassing difficult and time-consuming lab experiments. Ultimately, having multiple alleles that display the same phenotype allowed us to showcase both the fragility and the flexibility of *β*-propellers as sensor domains, properties that might be advantageous for immune receptors.

Classic experiments designed to inactivate *het* genes found that the HNWDs have much higher mutation rates than their binding partners, *het-c* and *het-v* [77]. Subsequent studies demonstrated that the HNWD genes are particularly susceptible to mutations at their sensor domain, altering or inactivating their recognition specificities by losses, gains and shuffling of repeats [33,35, 73]. This ‘repeat instability’ led to the suspicion that HNWDs (and potentially all NLRs with HIC) might easily rearrange during somatic growth, driven by unequal crossing-overs and intra- or interparalogue recombinations [30,35]. Our comparison of over 1,000 individual repeats indicates that variation clusters essentially by gene of origin, but on occasion, repeat units from different genes can be very similar or identical, as exemplified by the case of *het-e* and *nwd6*. This observation suggests that, although intraparalogue recombination events dominate in terms of frequency, the interparalogue exchanges potentially contribute to NLR HIC repeat diversification. However, if this process occurs during vegetative propagation in the lab, it is too infrequent in the sequencing reads to influence the assembly graph. Detecting somatic repeat mutations may require extremely deep long-read sequencing efforts. Nonetheless, intra-thallus diversity in NLR repeats might still be subject to selection in nature, in particular if occasional variants happen to improve recognition of non-self. It has been suggested that fungal NLRs might have a general innate immune system function similar to that of plants and animals [78,79]. Immune system genes often display high diversity maintained by balancing selection, which is also a characteristic necessary for genetic systems controlling conspecific self–non-self-recognition (allorecognition) [80,81]. Heterokaryon incompatibility is a type of allorecognition, and NLRs might occasionally get co-opted as *het* genes in different fungal lineages [78]. From that perspective, the high mutational rate associated with HIC repeats might be advantageous for both the innate immune system and allorecognition functions.

The fact that the Wa63+ *het-e* allele contains 11 HIC repeats, the usual size of a functional allele, and occurs in three different strains, sampled in different places and years, suggested that this might not be a random spontaneous mutant allele. However, our transformation essays demonstrated that this allele does not trigger an incompatibility reaction with the most common *het-c* alleles. That leaves us with three possibilities: (1) the allele is truly nonfunctional and its frequency is maintained by genetic drift; (2) the allele can only recognize some rare *het-c* alleles that were not tested here; or (3) the allele can recognize another ligand, such as a pathogen-derived molecule. Theoretically, an NLR *het* gene could simultaneously retain an ancestral immune function, further contributing to the maintenance of genetic diversity [73,80]. Population and ecological studies on *Podospora* NLRs might help clarify this point.

Sequencing multiple versions of the same *het* alleles revealed a surprising diversity of repeat numbers and sequence combinations, implying that the mutational input can easily converge to the same phenotypes. The sequenced *E1* and *E3* alleles can either be 11 or 12 HIC-repeats long, while the one known *E2* allele has 10 HIC repeats. AlphaFold 3-predicted models were consistent with the idea that the WD40 domain of HNWD genes folds into two *β*-propellers and revealed the presence of cryptic repeats at the C-terminus. As several length differences between *het-e* variants of the same phenotype occur at the end of the HIC region, perhaps the second *β*-propeller can potentially be formed by six to eight blades (in combination with the cryptic repeats) and remain functional. In contrast with the flexibility observed in *het-e*, the 3 known functional alleles of *het-d* all have 11 HIC repeats, and a single repeat change in the second propeller completely inactivated the *het-d* allele of the strain Z. Likewise, the only known reactive allele of *het-r* has 11 HIC repeats [35]. One might speculate that the flexibility of *het-e* is related to the exact form of the cryptic repeats, which correspond to a region highly diverged between the HNWD paralogues.

Just as different *het-e* sequences can have the same binding affinities, our analyses indicate that *het-e* and *het-d* independently evolved the capacity to recognize *het-c*. In particular, we found that these two NLRs are not each other’s closest relatives in phylogenetic analyses and that the HIC repeat variation does not overlap. Although the function of related NLRs such as *hnwd1* and *hnwd3* is unknown, it seems unlikely that they act as heterokaryon incompatibility genes themselves, since there has been no indication of other undiscovered *het* genes segregating in *P. anserina* populations throughout decades of study. Thus, the *het-d/e* recognition phenotype constitutes a case of convergent evolution, as similarly observed in some plant NLRs [82]. In addition, our analysis confirms a trend apparent in earlier genetic studies, namely, that many of the natural *het-d* and *het-e* alleles are inactive (*d3* and *e4* alleles). In the original 16 French isolates, 6 and 12 strains were found to harbour inactive *het-e* and *het-d* alleles, respectively [28]. Similarly, we found that a significant proportion of the Wageningen isolates bear inactive *het-d* or *het-e* alleles. The frailty of the HIC WD40-sensor domain may explain the need for redundancy in the capacity of surveying *het-c* allelic variants, and hence, the convergent evolution of *het-d* and *het-e*.

The presence of highly similar repeats in the WD40 sensor domain of HNWD genes might be peculiar but is not a unique case for WD40 proteins. A large-scale screening of proteins with WD40 domains (not just NLRs) across the Tree of Life revealed that HIC happens most often in fungal and bacterial genomes [39]. Moreover, NLRs with other types of superstructure-forming domains, such as ankyrin, tetratricopeptide and HEAT repeats, are also known to have HIC in different fungal groups [1,12]. There is even a report of a leucine-rich repeat NLR with HIC in a sea urchin genome [83]. In all these cases, the size of these repeat types is very similar to those of the WD40 repeats, between 24 and 42 aa [84,86]. Therefore, the challenges we faced with the short-read assembly of HNWD alleles are likely to apply to other NLRs across various taxonomic groups.

## Conclusion

Long-read technologies have been instrumental in the correct assembly of plant and animal NLRs since their development (e.g. [87,88]). However, the study of fungal NLRs is relatively new, and most genomic resources used in previous analyses have been Illumina based [12], simply because most non-model species lack high-quality assemblies. Certainly, many aspects of NLRs can be fully studied from Illumina data, such as domain composition, diversity and phylogenetic relationships. However, functional molecular biology studies can only do so much without high-confidence sequences, as in the case of *het-d* and *het-e*. Despite the availability of short-read population genomic data, the allele frequencies of these HNWD genes remain unknown. Such a gap hinders the study of potential balancing selection forces acting on them [89]. The increased availability of long-read assemblies will not only address this limitation but will simultaneously allow for the study of other aspects of their biology. For example, the genomic location of an NLR might influence its epigenetic modifications or mutation load [90]. Looking forward, comparative studies of HNWD genes across populations and species, coupled with functional assays, may uncover novel roles for these genes beyond heterokaryon incompatibility. Additionally, integrating structural predictions with mutational analyses can clarify how *β*-propeller architecture contributes to the specificity of these immune receptors.

## Declaration of generative AI and AI-assisted technologies in the writing process

During the preparation of this work, the authors used ChatGPT to improve the flow and grammar of some parts of the manuscript. After using this tool, the authors reviewed and edited the content as needed and take full responsibility for the content of the published article.

## Supplementary material

10.1099/mgen.0.001442 Uncited Supplementary Material 1.

10.1099/mgen.0.001442 Uncited Supplementary Material 2.

## Abbreviations

DNA CS: DNA control sample
HIC: high internal conservation
HMW: high-molecular-weight
LA: LB agar
LB: Luria–Bertani
NBD: nt-binding and oligomerization domain
NLR: NOD-like receptor
ONT: Oxford Nanopore Technologies
PCA: principal component analysis
