# Supplementary material for: Reconstructing NOD-like receptor alleles with high internal conservation in Podospora anserina using long-read sequencing
Source: Microb Genom. 2025 Jul 2;11(7):001442. doi: 10.1099/mgen.0.001442 (PMC12222747; doi:10.1099/mgen.0.001442)
Supplement: Uncited Supplementary Material 1. [file mgen-11-01442-s001.pdf]

Supplementary Figures of

Reconstructing NOD-like receptor alleles with high internal conservation in *Podospora anserina* using long-read sequencing

S. Lorena Ament-Velásquez, Brendan Furneaux, Sonia Dheur, Alexandra Granger-Farbos, Rike Stelkens, Hanna Johannesson, Sven J. Saupe

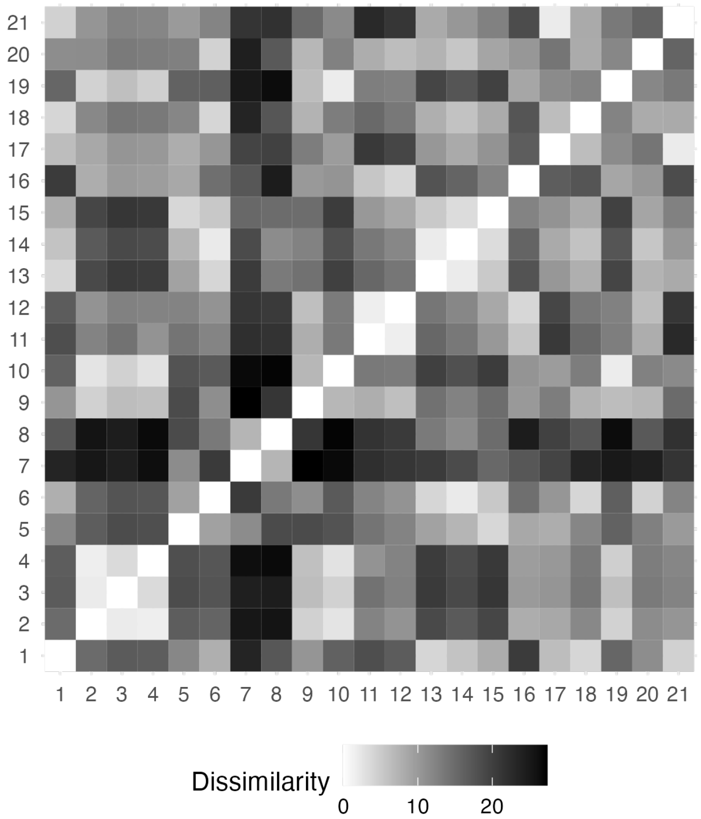

Figure S1. Heatmap of dissimilarity between the classes of HIC WD40 repeats of the *het-d* gene, based on seven amino acid positions.

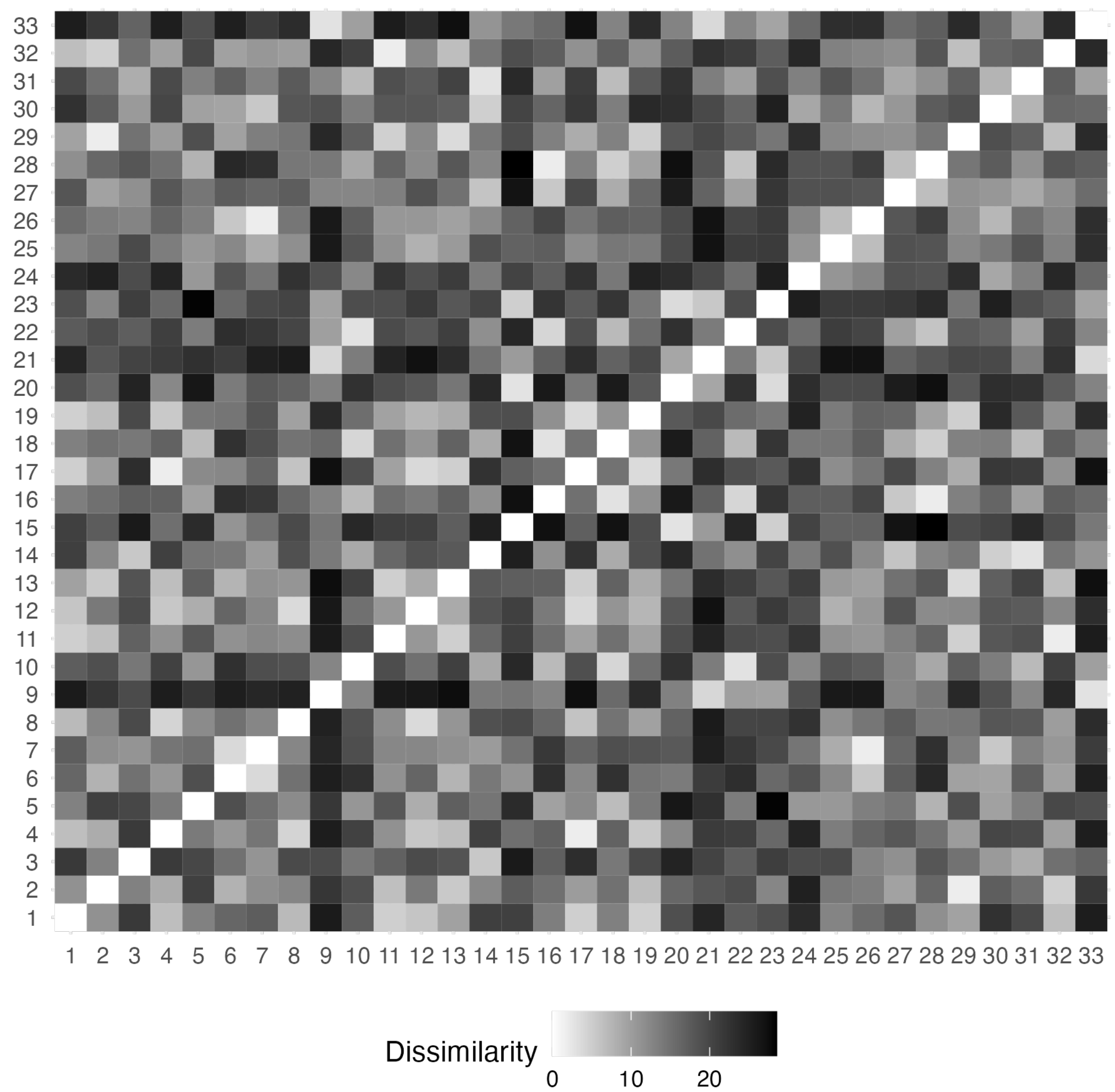

Figure S2. Heatmap of dissimilarity between the classes of HIC WD40 repeats of the *het-e* gene, based on seven amino acid positions.

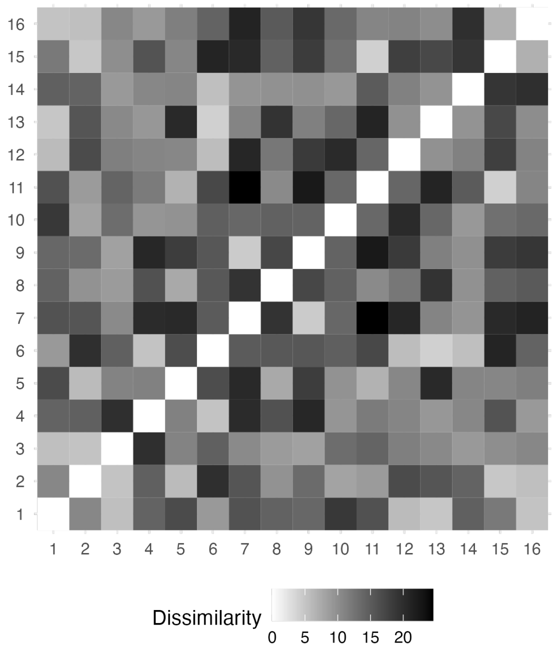

Figure S3. Heatmap of dissimilarity between the classes of HIC WD40 repeats of the *het-r* gene, based on seven amino acid positions.

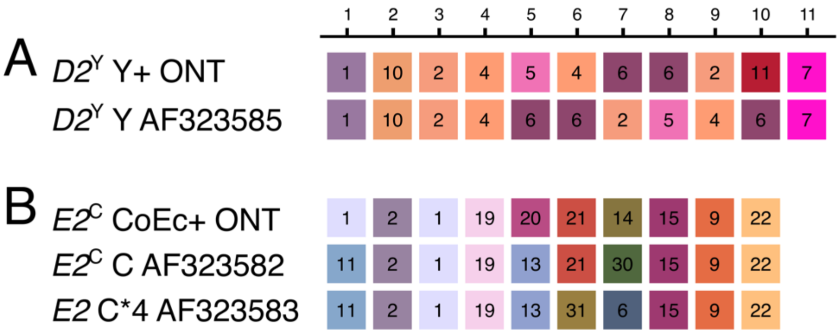

Figure S4. Comparison between the published alleles *D2*^{Y} (**A**) and *E2*^{C} (**B**) and corresponding long-read assemblies. The *E2* C*4 allele is a mutant of the original *E2*^{C} allele as reconstructed in the original study of Espagne et al. (2002).

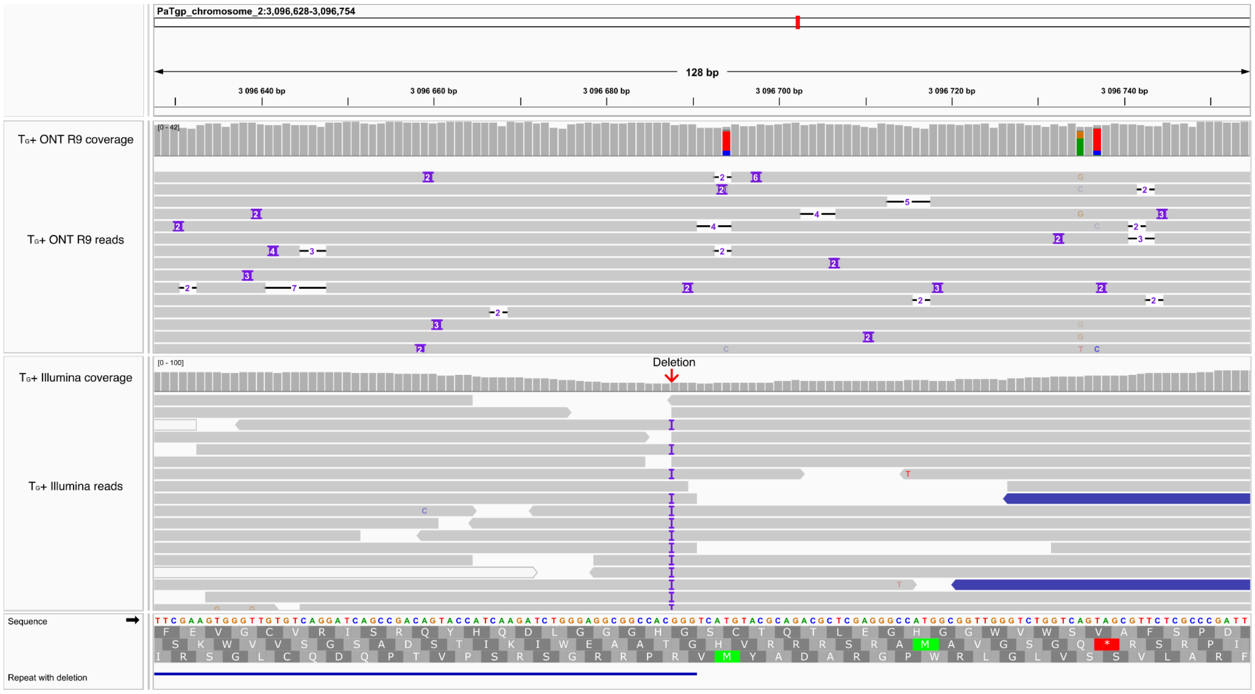

Figure S5. Short- and long-read mapping of the strain TG+ displayed in the Integrative Genomics Viewer (IGV) browser. Purple marks signal indels. Although not apparent in the long reads, the short-read mapping is consistent with a missing G (marked with a red arrow) at the end of the third repeat in the WD40 domain of *het-d*. White reads have multiple mappings. Blue reads signal smaller than expected insert size given the distribution of the paired-end reads.

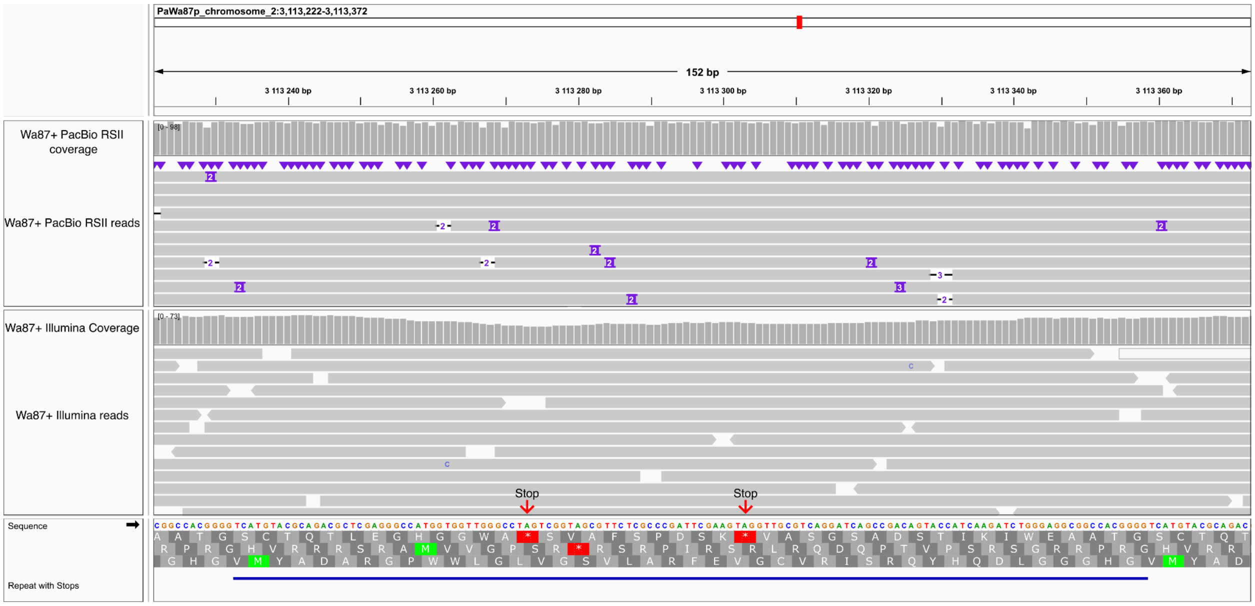

Figure S6. Short- and long-read mapping of the strain Wa87+ displayed in the Integrative Genomics Viewer (IGV) browser. Purple marks signal indels. The two stop codons found in the 6th repeat of the WD40 domain of *het-d* are marked with red arrows.

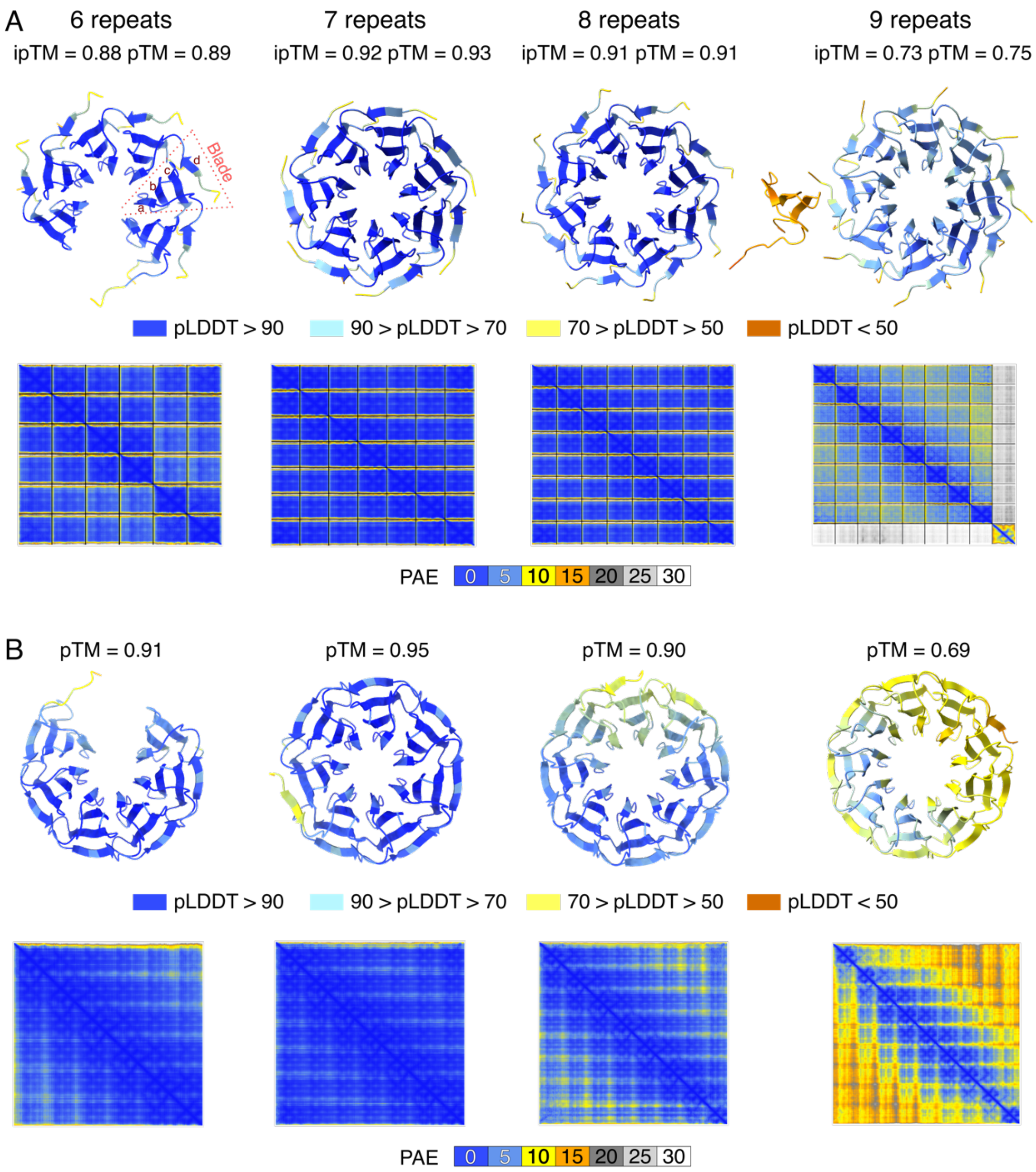

Figure S7. Ribbon diagrams of the β-propellers produced by AlphaFold 3 when different iterations of a HET-E2 repeat (second HIC repeat in the *E2*^{C} allele) are given. In (**A**) the individual repeat is input as multiple molecules to form a protein complex, while in (**B**) an artificial sequence with a given number of identical repeats folds into a single structure. The pLDDT score has a 0-100 scale where a higher value indicates higher confidence. The predicted template modeling (pTM) score and the interface predicted template modeling (ipTM) score have a scale from 0 to 1 and measure the accuracy of the entire structure (a score of 1 is best). The ipTM score in particular measures the accuracy of the relative positions of the subunits in the protein complex (in this case the blade monomers). The predicted aligned error (PAE) plots below the structures depict the error estimate in the relative position and orientation between residues (in angstroms); higher values indicate higher predicted error. An individual blade is formed by a *d* β-sheet from one repeat and the *a*, *b*, and *c* β-sheets of the next repeat, as highlighted in the first diagram of (**A**).

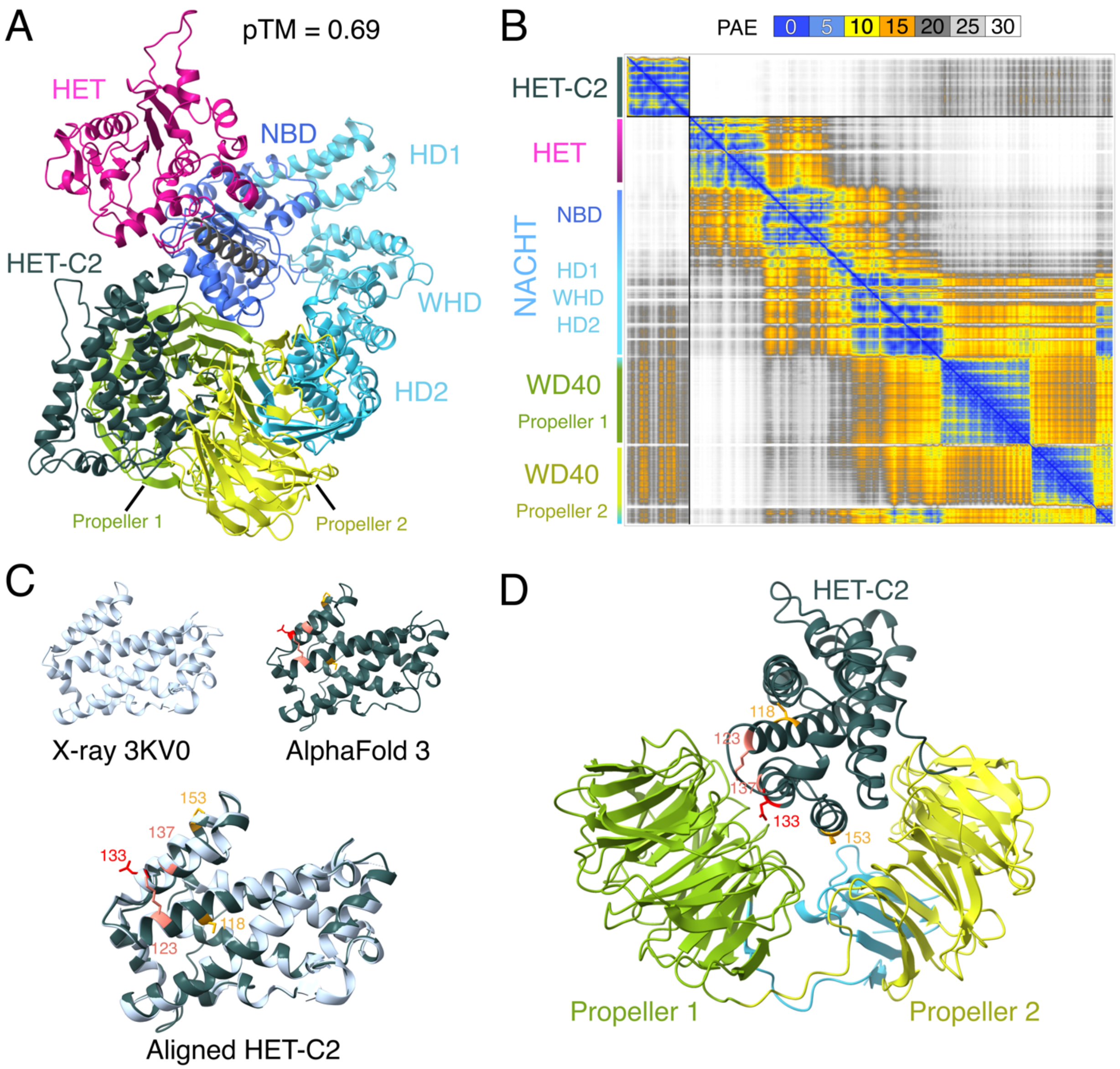

Figure S8. Tentative model of the HET-C2/HET-E1^{H} protein complex. In (**A**), the AlphaFold 3 model of HET-E1^{H} interacting with HET-C2 is displayed as a ribbon diagram. The corresponding predicted aligned error (PAE) plot depicting the error estimate in the relative position and orientation between residues in angstroms is shown in (**B**). The NACHT domain is composed of three subdomains: the nucleotide-binding domain (NBD), the helical domain 1 (HD1), the winged-helix domain (WHD), and helical domain 2 (HD2). The AlphaFold 3 HET-C2 structure in this model is congruent with the experimental X-ray structure (**C**). In the protein complex, HET-C2 is placed in between the WD40 propellers (**D**) (other domains were removed for clarity). The HET-C2 residues under positive selection (123, 133, and 137) and those known to be involved in allele specificity (118, 133, and 153) are highlighted with a stick representation in (**C**) and (**D**). pTM: predicted template modeling score.

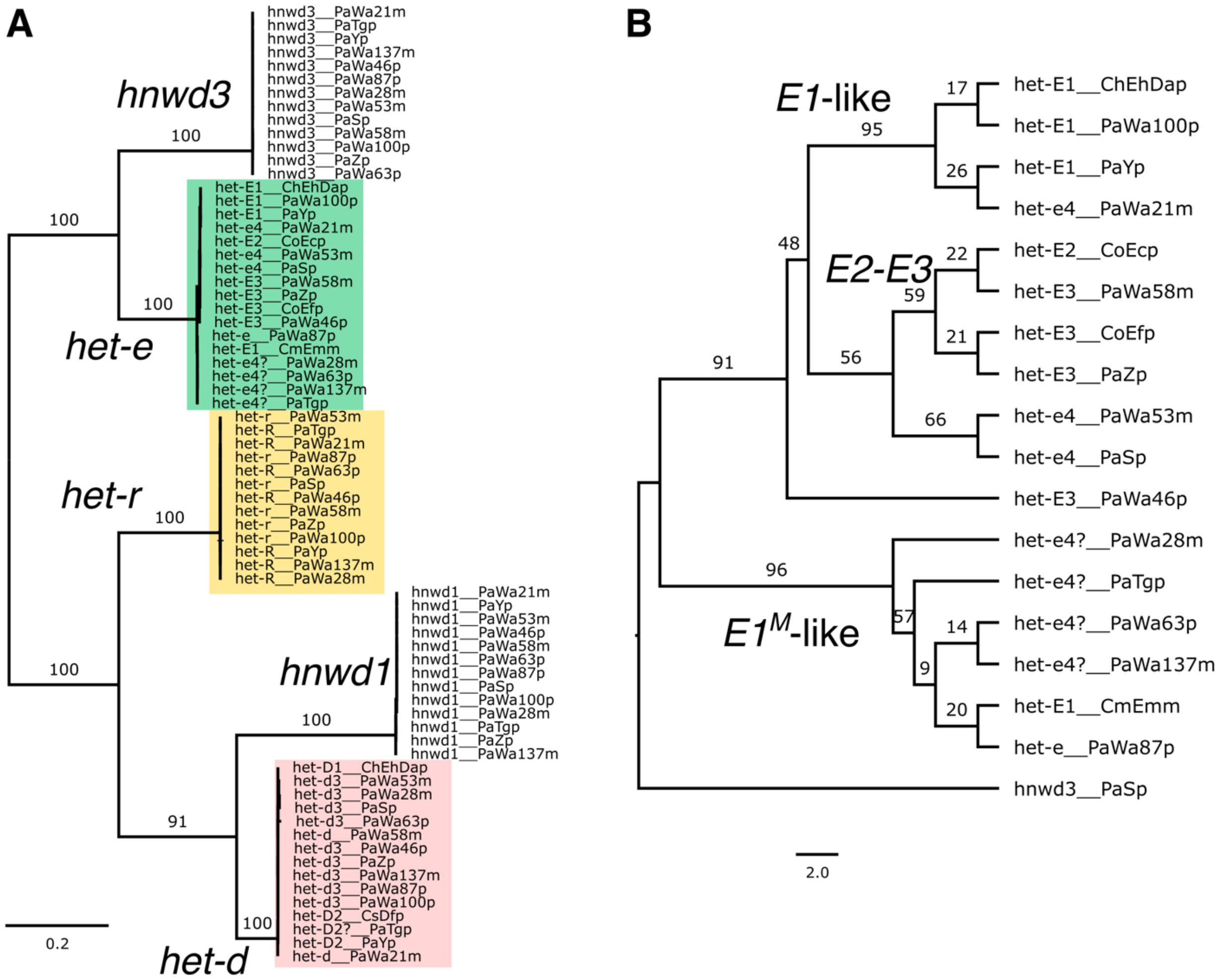

Figure S9. Phylogeny of the HNWD genes based on the HET and NACHT domains. (**A**) Maximum likelihood phylogeny of the HNWD genes including the alleles of all strains. Branch support values corresponds to standard non-parametric bootstrap (values within gene clades omited for clarity). Branches are proportional to the scale bar (nucleotide substitutions per site). (**B**) Cladogram of just the *het-e* alleles using *hnwd3* as outgroup. Branch support values corresponds to standard non-parametric bootstrap (branch lengths transformed with the "proportional" option in FigTree). Clades containing particular functional alleles and similar sequences are marked. Other *het* genes lack any obvious supported structure within their clades (not shown).

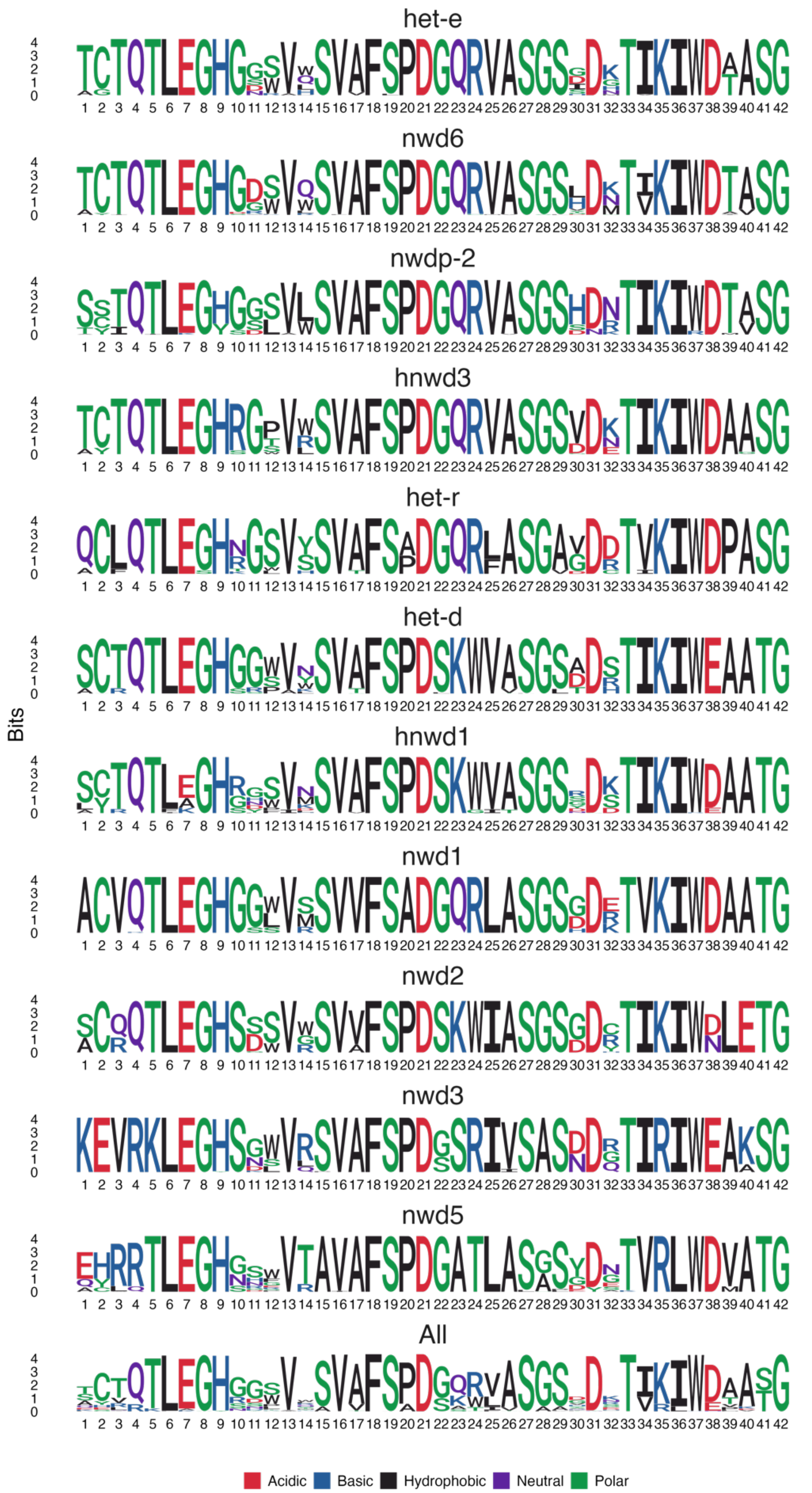

Figure S10. WD40 logo of the HNWD genes and related NLRs.

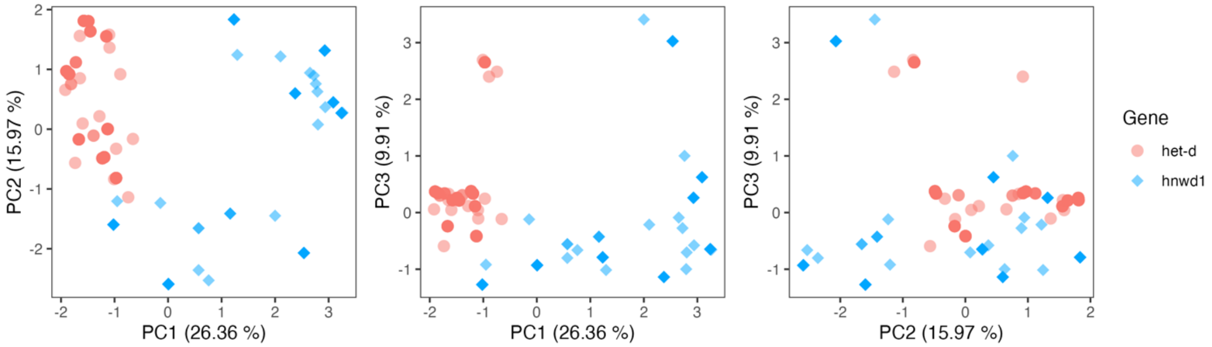

Figure S11. Principal component analysis of the *het-d* and *hnwd1* WD40 repeats with HIC.
